# Sustainable design of jewelry materials through green polymer and low impact processing strategies

**DOI:** 10.3389/fchem.2026.1856406

**Published:** 2026-09-03

**Authors:** Qiong Wang, Dengdeng Li, Na Guo

**Affiliations:** 1 College of Jewelry, Shanghai Jianqiao University, Shanghai, China; 2 Faculty of Humanities and Arts, Macau University of Science and Technology, Macau, China; 3 Architecture and Environment Art, Shanghai Urban Construction Vocational College, Shanghai, China

**Keywords:** green polymers, jewelry materials, low impact processing, SpectralSemanticTopology, sustainable design

## Abstract

**Introduction:**

The sustainable design of jewelry materials has become a critical focus in the pursuit of environmental conservation and responsible manufacturing practices. Traditional approaches to jewelry grade polymer development often prioritize aesthetic and structural qualities while neglecting environmental considerations, leading to significant ecological footprints and limited adaptability to green manufacturing standards. This study introduces SpectralSemanticTopology, a novel methodological framework that integrates advanced green polymer systems with low impact processing strategies to address these challenges.

**Methods:**

The framework is structured around three core modules: the Spectral Consistency Mapper, the Semantic Transition Checker, and the Topology Aware Trajectory Resolver, which collectively enable systematic exploration and optimization of polymer formulations and processing pathways. These modules are complemented by harmonic relation modeling and rule guided inference mechanisms, ensuring that the visual and tactile attributes essential to high end jewelry are preserved without compromising environmental sustainability. The proposed method formalizes the design problem within a rigorous mathematical framework, facilitating precise modeling and optimization of material properties and processing techniques.

**Results and Discussion:**

Experimental results demonstrate significant improvements in environmental performance metrics, including reductions in energy consumption and waste generation, while maintaining the aesthetic and structural integrity required for luxury applications. The findings underscore the potential of SpectralSemanticTopology to redefine sustainable practices in jewelry design, offering a pathway to innovative and environmentally responsible material development.

## 1 Introduction

The sustainable design of jewelry materials has become increasingly important in the context of environmental conservation and resource management. Not only does the jewelry industry contribute significantly to environmental degradation through mining and processing activities, but it also poses ethical concerns related to labor practices and resource exploitation. Moreover, the demand for eco friendly products is rising among consumers who are becoming more environmentally conscious. This necessitates the exploration of sustainable alternatives that minimize ecological impact while maintaining aesthetic and functional qualities. Green polymers and low impact processing strategies offer promising solutions to these challenges, enabling the creation of jewelry that is both beautiful and sustainable. By integrating these innovative materials and methods, the industry can not only reduce its carbon footprint but also enhance its appeal to a growing market of eco conscious consumers Geng (2024).

Early efforts to incorporate sustainability into jewelry design relied on manually analyzing material properties and environmental impacts. These methods often involved predefined frameworks to evaluate the ecological footprint of various materials, enabling designers to make informed choices. While these approaches provided a structured way to assess sustainability, they were constrained by their inability to adapt to evolving environmental data and consumer preferences Wannarumon and Bohez (2006). The static nature of these methods limited their effectiveness in addressing the dynamic challenges posed by sustainable design, prompting researchers to explore more adaptive strategies Chu (2022).

To enhance adaptability, researchers began employing computational techniques capable of analyzing diverse datasets. Algorithms were developed to evaluate material properties, production processes, and consumer trends, offering a more comprehensive approach to sustainable jewelry design. These methods allowed designers to identify optimal combinations of materials and techniques that minimized environmental impact while meeting aesthetic standards Wang et al. (2018). However, the reliance on extensive data processing introduced challenges related to computational efficiency and the interpretability of results, which sometimes hindered practical implementation He and Hao (2017).

Recent advancements have focused on leveraging sophisticated models capable of learning directly from complex datasets. Techniques such as neural networks have been applied to analyze intricate relationships between material properties and environmental factors, providing designers with actionable insights. Pre trained models have further enhanced this process by enabling knowledge transfer across domains, reducing the need for extensive data collection and training Wei (2023). Despite their promise, these methods require significant computational resources and careful calibration to avoid issues like overfitting. Nonetheless, their ability to balance precision and adaptability marks a pivotal step forward in achieving sustainable jewelry design Zhang et al. (2020).

Despite these advances, an explicit methodological gap remains in sustainable jewelry material design. Existing studies usually address environmental assessment, visual appearance, mechanical reliability, or processing feasibility as separate issues, but they rarely provide a unified computational mechanism for modeling their interactions within the same design space. In technical terms, material descriptors, sustainability indicators, perceptual attributes, and structural constraints are often optimized independently, which makes it difficult to evaluate trade offs among carbon related impact, recyclability, optical appearance, tactile quality, durability, and manufacturability. This limitation is particularly important for polymer materials used in jewelry applications, because a greener material substitution may reduce environmental burden while also changing spectral response, surface quality, stiffness, or processing compatibility. The unresolved problem is not only the selection of environmentally preferable polymers, but also the construction of a verifiable optimization framework that can jointly represent environmental performance, aesthetic requirements, and structural feasibility under low impact processing constraints.

To address this gap, this study proposes SpectralSemanticTopology, a unified computational framework for sustainable polymer jewelry material design. The framework is designed to model and optimize three coupled dimensions of material performance: environmental performance, perceptual quality, and structural reliability. Instead of treating these dimensions as isolated objectives, SpectralSemanticTopology connects molecular structure representation, property related spectral behavior, design consistency, and topology aware structural regularity within a coordinated regression and screening pipeline. The proposed framework is evaluated through polymer property prediction on the Polymer Genome Dataset and the PI1M Dataset, and its effectiveness is examined through comparisons with Random Forest, XGBoost, MLP, GCN, ChemBERTa, and LightGBM, as well as component ablation experiments.

The main contributions of this study are summarized as follows.

- A unified SpectralSemanticTopology framework is developed for sustainable polymer jewelry material design, in which spectral consistency, semantic design consistency, and topology aware structural regularity are modeled as coordinated components rather than independent objectives.
- A sustainability oriented optimization strategy is introduced to support polymer property prediction and candidate screening under environmental, perceptual, and structural constraints. The framework combines harmonic relation modeling with rule guided inference to improve the consistency of material representation and the feasibility of sustainable material selection.
- Experimental validation is conducted on the Polymer Genome Dataset and the PI1M Dataset under a supervised regression setting. The proposed method is compared with six representative baselines, and ablation results are provided to verify the contribution of each functional component to prediction accuracy and model robustness.

## 2 Related work

### 2.1 Biodegradable polymers in jewelry design

The utilization of biodegradable polymers in jewelry design has emerged as a pivotal advancement in sustainable material science, offering a viable alternative to conventional materials due to their capacity for natural decomposition, which mitigates environmental impact Wei (2023). These polymers, including polylactic acid, polyhydroxyalkanoates, and polycaprolactone, are synthesized from renewable resources such as corn starch and sugarcane, significantly reducing the carbon emissions associated with their production Lin et al. (2019). Their application in jewelry design addresses critical issues of waste management while aligning with the increasing consumer preference for environmentally responsible products Zhang et al. (2020). The mechanical properties of these polymers are a key consideration, as they must exhibit sufficient strength and durability to endure daily use without compromising structural integrity Zhang (2010). Advances in polymer composites, such as the integration of natural fibers like bamboo into the polymer matrix, have enhanced their mechanical performance, making them suitable for intricate jewelry designs Han-li (2011). Aesthetic versatility is another essential attribute, as these materials can be engineered to display diverse colors, textures, and finishes, enabling designers to meet varied consumer preferences Jiang et al. (2025). Techniques such as 3D printing and injection molding have further expanded the creative possibilities by allowing precise fabrication of complex designs Aboagyewaa-Ntiri (2024). Sustainable processing methods, including solvent casting and melt extrusion, are employed to minimize energy consumption and maintain the biodegradability of the final product Ortega-Feliu et al. (2020). Research efforts continue to focus on optimizing polymer blends and processing techniques to improve the performance and sustainability of biodegradable materials in jewelry applications Zhang and Shen (2024).

### 2.2 Recycled metals and alloys

The incorporation of recycled metals and alloys into jewelry design represents a significant step toward sustainable material practices, as it reduces the environmental burden associated with mining and processing virgin materials Lyu et al. (2023). Precious metals such as gold, silver, and platinum are often recycled from post consumer waste, industrial scrap, and electronic components, providing a sustainable alternative to newly mined resources Rajili et al. (2014). The recycling process involves advanced techniques like electrolysis and chemical leaching to purify metal scrap, ensuring the production of high quality materials suitable for jewelry applications Ma et al. (2024). These methods are designed to minimize energy use and reduce the release of harmful byproducts, adhering to the principles of green chemistry Nervis and Selau (2023). Ethical concerns surrounding traditional mining practices, including labor exploitation and environmental degradation, are also addressed through the use of recycled metals, which align with the values of socially conscious consumers Jiaxin (2024). The development of recycled metal alloys, such as combinations of gold and silver or platinum and palladium, has expanded the material palette available to designers, offering enhanced properties like increased hardness and unique color variations Xia and Yao (2024). These alloys provide opportunities for innovation in sustainable jewelry design while maintaining high aesthetic and functional standards Tenuta et al. (2024). Ongoing research in metallurgical science aims to improve the efficiency of recycling processes and develop novel alloy compositions with superior properties, further advancing the integration of recycled metals in the jewelry industry Maţcan-Lîsenco (2025). The adoption of these practices not only reduces the environmental footprint of jewelry production but also supports ethical and sustainable approaches within the fashion sector Lerma et al. (2017).

### 2.3 Low impact processing techniques

Low impact processing techniques are integral to the sustainable production of jewelry, as they reduce energy consumption, minimize waste, and lower the overall environmental footprint of manufacturing processes Huang and Chen (2025). Additive manufacturing, commonly referred to as 3D printing, has revolutionized jewelry production by enabling the precise creation of complex designs with minimal material waste Boya and Hashim (2026). This method involves the layer by layer deposition of materials, allowing for the fabrication of customized pieces with high precision while reducing reliance on traditional, energy intensive casting methods Danso et al. (2026). The use of biodegradable polymers and recycled metals in additive manufacturing further enhances the sustainability of this approach Lin et al. (2019). Cold forging, which involves shaping metals at ambient temperatures, eliminates the need for energy intensive heating processes, thereby reducing energy consumption and enhancing the mechanical properties of the final product Zhang (2010). This technique is particularly effective for producing simple yet elegant designs with smooth finishes, achieved without the use of chemical treatments Jiang et al. (2025). Chemical free finishing methods, such as mechanical polishing and sandblasting, are also critical for sustainable jewelry production, as they avoid the use of toxic substances while achieving desired surface aesthetics Ortega-Feliu et al. (2020). These techniques contribute to the environmental integrity of the final product while maintaining its visual appeal Lyu et al. (2023). Research in this domain is focused on developing innovative methods that further reduce the environmental impact of jewelry manufacturing, leveraging advancements in material science and engineering to enhance the sustainability of production processes Ma et al. (2024). The implementation of low impact processing techniques not only supports environmental sustainability but also ensures the creation of high quality, ethically produced jewelry Jiaxin (2024).

### 2.4 Position relative to sustainable polymer design and AI based material selection

Recent studies on sustainable polymers emphasize that environmental performance should be evaluated from a lifecycle perspective, including raw material sourcing, production, use, waste management, and end of life treatment. For example, lifecycle assessment studies on bioplastics show that bio based or biodegradable characteristics alone are not sufficient to determine environmental superiority Ali et al. (2023), and that processing energy, waste treatment, carbon related impact, and disposal pathways should also be considered Yadav and Nikalje (2024). Sustainable polymer jewelry material design requires not only the selection of greener polymer candidates, but also the evaluation of processing feasibility, durability, recyclability, and long term use performance. Multi objective optimization has been widely used in material design to balance competing requirements such as performance, stability, cost, and environmental impact Yan et al. (2021). Conventional optimization methods usually rely on predefined objective functions and manually selected indicators. They are effective for trade off analysis, but they do not directly learn the coupled relationship among molecular structure, appearance related response, design suitability, and structural regularity. The proposed framework introduces spectral, semantic, and topological representations to jointly model these coupled factors within a trainable prediction and screening pipeline. AI based material design and polymer informatics have provided important tools for polymer property prediction and virtual material screening Chen et al. (2021). Recent studies have further extended polymer informatics from homopolymers to more complex polymer systems and composites Shukla et al. (2024), showing the potential of machine learning in predicting thermal, mechanical, electrical, and functional properties Tran et al. (2025). Different from general polymer property prediction models, the proposed SpectralSemanticTopology framework connects polymer structure encoding, supervised property prediction, and rule guided sustainable material recommendation. This design allows the predicted property values to support downstream screening under environmental, structural, processing, aesthetic, and tactile requirements for jewelry material applications.

Existing multi objective optimization methods usually define sustainability, mechanical performance, cost, and processing feasibility as separate objectives, and then search for Pareto optimal solutions. These methods are useful for trade off analysis, but they often depend on manually defined indicators and fixed objective settings. They do not explicitly model the coupled relationship among polymer molecular structure, appearance related response, design suitability, and structural regularity. Sustainable material design studies mainly focus on environmental assessment, lifecycle comparison, recyclability, biodegradability, and low impact manufacturing. These studies provide important evaluation criteria, but many of them are applied after candidate materials have been selected. The proposed framework uses supervised polymer property prediction as an early screening step and further combines the predicted results with sustainability and processing constraints. AI based material selection methods, such as descriptor based learning, graph neural networks, and pretrained molecular models, can predict material properties from molecular structures. Most of them focus on numerical property prediction and do not explicitly connect the prediction result with jewelry grade perceptual quality, sustainability rules, and manufacturability constraints. The proposed SpectralSemanticTopology framework differs by learning coupled spectral, semantic, and topological representations, and by integrating property prediction with rule guided sustainable material recommendation.

## 3 Methods

In this study, green polymers refer to polymer materials that can reduce environmental burden during material sourcing, processing, use, or end of life treatment while maintaining the functional requirements of jewelry applications. Green polymers include bio based polymers, biodegradable polymers, recyclable polymers, polymers with reduced toxicity risk, and polymers compatible with low energy or low waste processing routes. This definition does not imply that a polymer is environmentally optimal in all lifecycle stages. It indicates that the polymer shows at least one measurable environmental advantage, such as renewable content, recyclability, biodegradation potential, lower processing energy, lower material loss, or improved end of life feasibility, compared with conventional polymer alternatives. Under this definition, a polymer candidate is not accepted as a green jewelry material only because it belongs to a bio based or biodegradable category. It must also satisfy application related constraints, including mechanical reliability, dimensional stability, surface quality, aesthetic stability, tactile acceptability, and processing compatibility. Green polymers are treated in this study as sustainability oriented candidates that require further prediction, constraint filtering, and fabrication oriented validation before final recommendation.

### 3.1 Overview

This section outlines the methodological framework for the sustainable design of jewelry materials, focusing on green polymer systems and low impact processing strategies. The proposed SpectralSemanticTopology framework is implemented as a supervised polymer property prediction and constraint based material screening pipeline. It aims to jointly evaluate environmental performance, aesthetic attributes, and structural reliability of jewelry grade polymers under realistic manufacturing constraints. In technical terms, the framework consists of three representation learning components. The spectral component learns property aligned and appearance related representations, the semantic component regularizes design suitability, sustainability preference, tactile acceptability, and processing compatibility, and the topological component preserves molecular connectivity and structural coherence through graph based regularization. These components are further integrated through feature fusion and rule guided candidate filtering, which support the screening of green polymer formulations and low impact processing routes while maintaining the visual and tactile qualities required for high end jewelry applications.

The problem is formalized as a constrained mapping from a space of green polymer compositions and processing parameters to a multi dimensional performance space encompassing environmental indicators, mechanical properties, and perceptual qualities. Jewelry materials are represented as structured entities encoding spectral responses, semantic level design attributes, and topological characteristics of both microstructure and macroscopic form. The SpectralSemanticTopology framework is defined as a triplet of coupled latent spaces: a spectral space capturing optical and colorimetric behavior, a semantic space encoding design intent and usage context, and a topological space describing geometric and connectivity properties of the material and artifact. To make the sustainability objective operational, the variables considered in the framework are divided into three groups: aesthetic variables, structural variables, and sustainability variables. Aesthetic variables describe perceptual and appearance related properties, including spectral response, color stability, surface gloss, brilliance, transparency, fluorescence, surface texture, and tactile perception. Structural variables describe material reliability and manufacturing feasibility, including molecular connectivity, graph level regularity, pore continuity, surface roughness, stiffness, toughness, dimensional stability, and process induced structural stability. Sustainability variables describe environmental and lifecycle related factors, including carbon related impact, renewable or bio based content, recyclability, processing energy, curing or post processing demand, material loss, water consumption, fabrication emissions, repairability, durability, reuse potential, and end of life processing feasibility. The spectral and topological components are not used as direct environmental impact estimators. Their role is to preserve feasibility when sustainability oriented material substitution is performed. Aesthetic variables ensure that greener polymer candidates remain acceptable in visual and tactile quality, while structural variables ensure that these candidates remain mechanically reliable and manufacturable. Sustainability variables provide the explicit environmental and processing criteria used for scoring and filtering. Therefore, the framework treats sustainable jewelry material design as a coupled optimization problem, in which environmental preference must be considered together with appearance preservation and structural feasibility. A candidate with lower environmental burden may still be unsuitable if it fails to meet jewelry grade perceptual quality, durability, or manufacturability requirements.

The Spectral Consistency Mapper relates polymer composition and processing parameters to spectral signatures, including reflectance, transmittance, and fluorescence, which influence perceived color, brilliance, and depth. The Semantic Transition Checker evaluates whether material and process configurations remain coherent with the original design intent, ensuring that transitions toward bio based polymers or low energy curing routes do not compromise tactile feel, perceived durability, or visual appeal. The Topology Aware Trajectory Resolver models the geometric and structural aspects of jewelry artifacts, capturing process induced changes in topology, such as pore connectivity and surface roughness, which are critical for both mechanical robustness and optical effects. The strategy layer incorporates harmonic relation modeling, which emphasizes balance across spectral, semantic, and topological variables, and rule guided inference, which encodes domain specific constraints and preferences to steer the search within the configuration space. Together, these components enable the identification of sustainable design pathways that align with both technical feasibility and aesthetic expectations.

### 3.2 Preliminaries

This section establishes the mathematical framework for integrating sustainable materials and low impact processing strategies in jewelry design. The objective is to model the interactions between material properties, environmental impact, and design constraints, ensuring that the resulting designs meet both aesthetic and functional requirements while minimizing environmental harm. The formulation is designed as an operational basis for the SpectralSemanticTopology framework rather than as an abstract symbolic description. Each variable used in the following equations corresponds to a computable quantity derived from polymer molecular structures, material descriptors, processing parameters, or sustainability constraints.

Before defining the optimization objective, three operational spaces are introduced. The spectral space represents measurable appearance related and property related responses, including optical behavior, color stability, surface response, gloss, transparency, thermal stability, and other indicators associated with polymer performance. These variables are obtained from available material measurements, molecular descriptors, and normalized property indicators. In implementation, a material is mapped to a spectral latent vector $s\in R^{d_{s}}$ through the Spectral Consistency Mapper. The corresponding output is a spectral consistency score that indicates whether the candidate polymer preserves the required visual and surface characteristics. This property aligned representation is consistent with materials informatics studies that connect molecular structure descriptors with target material properties Ramakrishna et al. (2019); Butler et al. (2018). The semantic space represents design level suitability, including aesthetic consistency, tactile acceptability, sustainability preference, processing compatibility, and suitability for jewelry use. The semantic variables are derived from design labels, expert ratings, text descriptions, and material attribute vectors. Design labels may describe transparency, brilliance, surface texture, tactile softness, perceived durability, product type, and intended use. Expert ratings are normalized to represent aesthetic quality, tactile quality, and application suitability, while textual design descriptions can be converted into numerical embeddings through a language encoder. Sustainability attributes, including recyclability, bio based content, processing temperature, and end of life feasibility, can also be included in the semantic representation. Design intent is quantified as a target attribute vector containing the desired levels of these design and sustainability variables. For example, a lightweight translucent jewelry component can be encoded with high target values for transparency, surface smoothness, tactile comfort, recyclability, and compatibility with low temperature processing. The semantic latent vector $z\in R^{d_{z}}$ is generated from descriptor based, graph based, and token based molecular representations. The Semantic Transition Checker compares this vector with the target design intent and outputs a semantic compatibility score. The topological space represents molecular connectivity and structural regularity. It is implemented through the molecular graph G=(V,E) , where atoms are treated as nodes and chemical bonds are treated as edges. Node variables include atom type, valence, aromaticity, formal charge, and hybridization, while edge variables include bond type, bond order, and conjugation state. The learned topological representation is denoted as $g\in R^{d_{g}}$ . In this study, a topological trajectory denotes the sequence of latent structural states generated during representation updating and candidate screening, rather than a physical motion path. The Topology Aware Trajectory Resolver outputs a structural regularity score that reflects molecular coherence and processing feasibility. Graph based molecular modeling provides the theoretical basis for using such topological representations in polymer property prediction Poelking et al. (2018); Audus and de Pablo (2017). The three spaces therefore provide complementary and interpretable outputs. For each polymer candidate, the framework produces a predicted material property value, a spectral consistency score, a semantic compatibility score, and a topological feasibility score. These outputs are combined with sustainability and processing constraints to rank and filter candidate materials before physical prototyping, thereby connecting the learned representations with practical jewelry material selection and design decision making.

**FIGURE 1:**
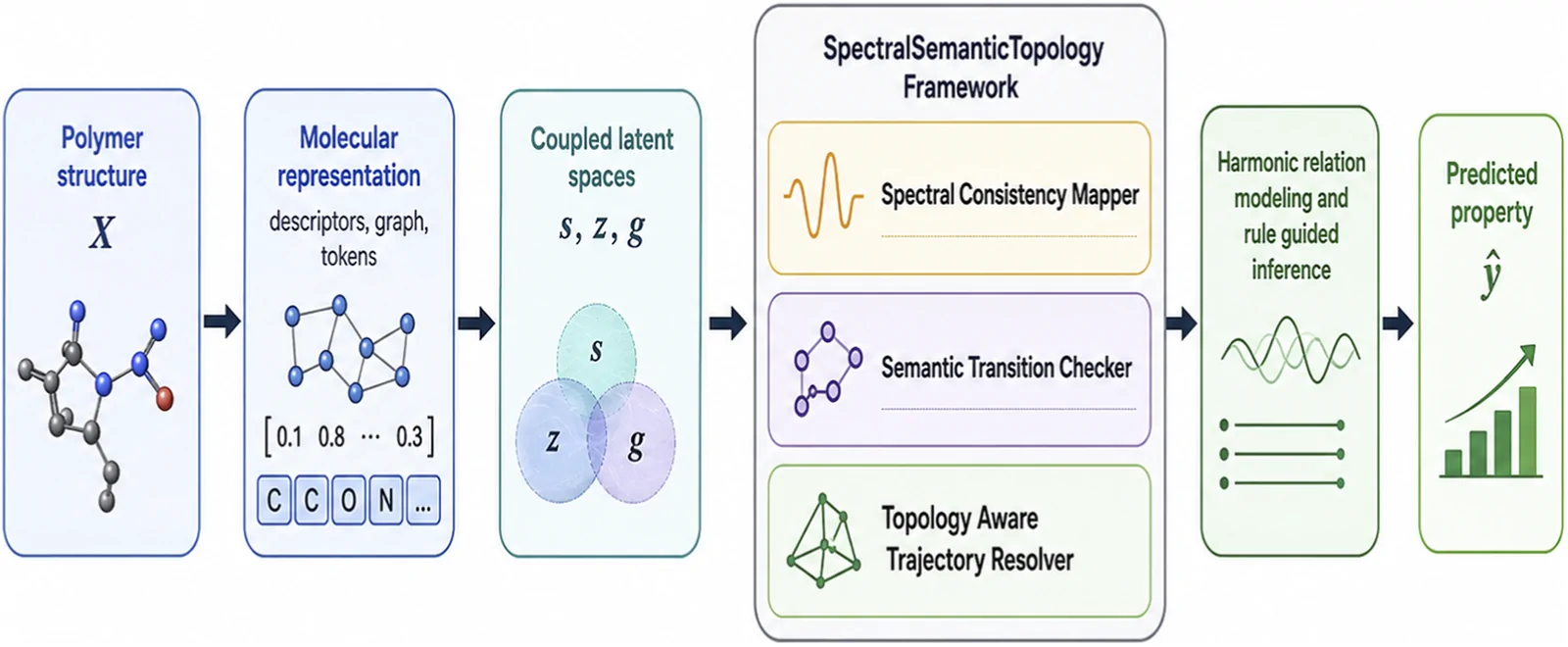
Variable mapping in the SpectralSemanticTopology framework. Polymer structures are transformed into descriptor based, graph based, and token based molecular representations, which are then projected into coupled spectral, semantic, and topological latent spaces. The three functional modules process these latent representations, and harmonic relation modeling with rule guided inference integrates the outputs for final polymer property prediction.

Figure 1 illustrates the variable mapping process of the SpectralSemanticTopology framework. The polymer structure X is first transformed into molecular representations, including molecular descriptors, graph representations, and tokenized structure sequences. These representations are then projected into the coupled latent spaces s , z , and g , corresponding to spectral, semantic, and topological representations, respectively. The three latent representations are processed by the Spectral Consistency Mapper, the Semantic Transition Checker, and the Topology Aware Trajectory Resolver. Their outputs are further integrated through harmonic relation modeling and rule guided inference, and the final regression head produces the predicted polymer property value $\hat{y}$ . This mapping clarifies how the abstract variables used in the mathematical formulation are connected to the implemented training and inference pipeline.

Let M denote the set of all candidate materials, where each material m∈M is described by a vector of properties $p_{m}=\left(p_{1},p_{2},\ldots ,p_{n}\right)$ . These properties include mechanical strength, thermal stability, and environmental metrics such as carbon footprint and recyclability. The environmental impact of a material m is represented by E(m) , which is modeled as a linear function of its properties (Equation 1):

$$E\left(m\right)=f\left(p_{m}\right)=\overset{n}{\underset{i=1}{\sum }}\alpha_{i}p_{i},$$ (1)

where $\alpha_{i}$ are coefficients that quantify the relative contribution of each property $p_{i}$ to the overall environmental impact. This formulation follows a weighted indicator aggregation principle that is commonly used in sustainable material assessment and material selection, where heterogeneous indicators are normalized and combined into a comparable score. In this study, each property $p_{i}$ is normalized using training subset statistics before aggregation, and $\alpha_{i}$ represents the importance weight assigned to the corresponding property or sustainability indicator. Therefore, E(m) is used as a relative material level environmental score for comparison, regularization, and candidate screening rather than as a complete life cycle assessment value Awasthi et al. (2011); Zavadskas et al. (2016).

To better capture sustainability objectives in polymer based jewelry manufacturing, the material level score is extended by considering process level, lifecycle level, and end of life level factors. Sustainable polymer manufacturing depends not only on intrinsic material properties, but also on processing energy, curing or post processing requirements, support material waste, water consumption, fabrication emissions, durability, repairability, and end of life treatment. The expanded sustainability score is defined as Equation 2:

$$E_{\text{sus}}\left(m,s\right)=E_{\text{mat}}\left(m\right)+E_{\text{proc}}\left(m,s\right)+E_{\text{life}}\left(m\right)+E_{\text{eol}}\left(m\right),$$ (2)

where $E_{\text{mat}}\left(m\right)$ denotes the material level sustainability score, $E_{\text{proc}}\left(m,s\right)$ denotes the process level sustainability score associated with processing strategy s , $E_{\text{life}}\left(m\right)$ denotes the lifecycle performance score, and $E_{\text{eol}}\left(m\right)$ denotes the end of life feasibility score. These terms are computed from normalized indicators as follows Equations 3, 4:

$$E_{\text{mat}}\left(m\right)=\overset{A}{\underset{a=1}{\sum }}\eta_{a}u_{a}\left(m\right),\;E_{\text{proc}}\left(m,s\right)=\overset{B}{\underset{b=1}{\sum }}\rho_{b}v_{b}\left(m,s\right),$$ (3)

$$E_{\text{life}}\left(m\right)=\overset{C}{\underset{c=1}{\sum }}\mu_{c}r_{c}\left(m\right),\;E_{\text{eol}}\left(m\right)=\overset{D}{\underset{d=1}{\sum }}\xi_{d}h_{d}\left(m\right).$$ (4)

In these expressions, $u_{a}\left(m\right)$ represents material level indicators, including carbon related impact, renewable or bio based content, toxicity risk, recyclability, and biodegradation potential. The variable $v_{b}\left(m,s\right)$ represents process level indicators, including processing energy requirement, curing or post processing demand, thermal processing constraint, support material waste, water consumption, solvent demand, fabrication emissions, and manufacturing method dependence. The variable $r_{c}\left(m\right)$ represents lifecycle indicators, including predicted durability, wear resistance, repairability, and probability of premature failure. The variable $h_{d}\left(m\right)$ represents end of life indicators, including mechanical recycling feasibility, chemical recycling feasibility, reuse potential, compostability, and disposal burden. The coefficients $\eta_{a}$ , $\rho_{b}$ , $\mu_{c}$ , and $\xi_{d}$ control the relative importance of the corresponding indicator groups. All indicators are normalized before aggregation so that variables measured in different units can be compared within the same screening process. In implementation, directly measured sustainability indicators are used when they are available in the data source or material profile. For public polymer datasets where complete lifecycle and manufacturing measurements are unavailable for every candidate, missing factors are represented by normalized proxy indicators or rule guided constraints during candidate screening. Processing energy, waste generation, durability, repairability, and end of life feasibility enter the framework as sustainability constraints and filtering criteria after property prediction rather than as unobserved supervised labels. This design allows the framework to incorporate concrete sustainability considerations while remaining consistent with the available polymer informatics data.

The design process involves selecting a subset of materials S⊆M that minimizes the total environmental impact while satisfying a set of design constraints. These constraints are expressed as Equation 5:

$$g_{i}\left(p_{m}\right)\leq b_{i},\;\forall m\in S,$$ (5)

where $g_{i}$ are constraint functions, and $b_{i}$ are the corresponding bounds imposed by the design requirements. The constraint function $g_{i}\left(p_{m}\right)$ represents a measurable requirement imposed on a candidate polymer, such as minimum mechanical strength, acceptable thermal stability, recyclability threshold, or processing compatibility. The bound $b_{i}$ is determined by the corresponding jewelry grade design requirement or sustainability rule. In implementation, these constraints are used during candidate screening to remove materials that show favorable predicted properties but fail to satisfy basic environmental, structural, or processing feasibility requirements Gilmer et al. (2017); Duvenaud et al. (2015).

To account for the influence of processing strategies, we introduce a processing function P(m,s) , which quantifies the environmental impact of applying a specific processing strategy s to material m . The set of available processing strategies is denoted by $S_{p}$ , where each strategy $s\in S_{p}$ is characterized by a vector $q_{s}=\left(q_{1},q_{2},\ldots ,q_{k}\right)$ . The processing impact is defined as Equation 6:

$$P\left(m,s\right)=\overset{k}{\underset{j=1}{\sum }}\beta_{j}q_{j},$$ (6)

where $\beta_{j}$ are coefficients that represent the influence of each processing parameter $q_{j}$ on the overall impact. The processing vector $q_{s}$ contains normalized process variables, including processing temperature, curing time, energy demand, material loss rate, solvent requirement, and compatibility with additive manufacturing or low temperature processing. The coefficient $\beta_{j}$ controls the contribution of each processing variable to the processing impact score. This formulation follows process based sustainability evaluation, in which manufacturing parameters are converted into comparable indicators for low impact production assessment.

The total impact T(m,s) of using material m with processing strategy s is the sum of the material impact E(m) and the processing impact P(m,s) (Equation 7):

$$T\left(m,s\right)=E\left(m\right)+P\left(m,s\right).$$ (7)

This joint impact term links intrinsic material sustainability with processing sustainability. This combination is necessary because a polymer with favorable molecular or environmental characteristics may still be unsuitable for sustainable jewelry production when it requires high energy processing, intensive solvent treatment, or excessive material waste. Thus, T(m,s) provides a joint material and process score for subsequent optimization and screening.

The goal is to minimize T(m,s) over all combinations of materials and processing strategies, subject to the design constraints (Equations 8, 9):

$$\underset{m\in S,s\in S_{p}}{\min}T\left(m,s\right),$$ (8)

$$\text{subject to }g_{i}\left(p_{m}\right)\leq b_{i},\;\forall m\in S.$$ (9)

In practical implementation, the constrained optimization is realized through a supervised regression and candidate screening pipeline. Each candidate material m is represented by a canonical polymer molecular structure. The property vector $p_{m}$ is constructed from molecular descriptors, graph features, learned structure embeddings, and available sustainability related indicators. Each processing strategy s is represented by the normalized vector $q_{s}$ . During model training, the polymer structure is mapped to spectral, semantic, and topological latent representations, and the regression model predicts the target polymer property value $\hat{y}$ . The model is optimized by minimizing the difference between $\hat{y}$ and the recorded dataset label. During inference, candidate polymers are first ranked by predicted property value and then filtered according to E(m) , P(m,s) , and $g_{i}\left(p_{m}\right)\leq b_{i}$ . Therefore, the mathematical formulation defines the decision logic of the implemented screening procedure.

To solve this optimization problem, we employ a computational framework that integrates spectral, semantic, and topological analyses. The first module is the Spectral Consistency Mapper. It maps the molecular representation of a polymer into the spectral latent vector s , which captures property related and appearance related responses. This module evaluates whether the learned material representation is consistent with the target property profile and sustainability oriented appearance requirements. The second module is the Semantic Transition Checker. It maps the material representation into the semantic latent vector z , which encodes design suitability, tactile consistency, sustainability preference, and processing compatibility. This module checks whether a transition from a conventional material option to a greener polymer candidate preserves the functional and aesthetic requirements of jewelry design. The third module is the Topology Aware Trajectory Resolver. It operates on the molecular graph representation and produces the topological latent vector g . This module models atom bond connectivity, graph level structural regularity, and process related feasibility. The topological trajectory is implemented as the evolution of latent graph states during representation learning and candidate optimization. The integration of these modules ensures that the selected materials and processing strategies adhere to sustainable design principles while maintaining the desired quality and functionality of the jewelry. The three module outputs are fused by harmonic relation modeling and constrained by rule guided inference. The fused representation is then passed to the regression head for polymer property prediction. This computational procedure connects the variables in the mathematical formulation with the actual training and inference workflow, improving the reproducibility and interpretability of the proposed framework. This formalization provides a foundation for developing innovative approaches to environmentally responsible jewelry design.

### 3.3 SpectralSemanticTopology

**FIGURE 2:**
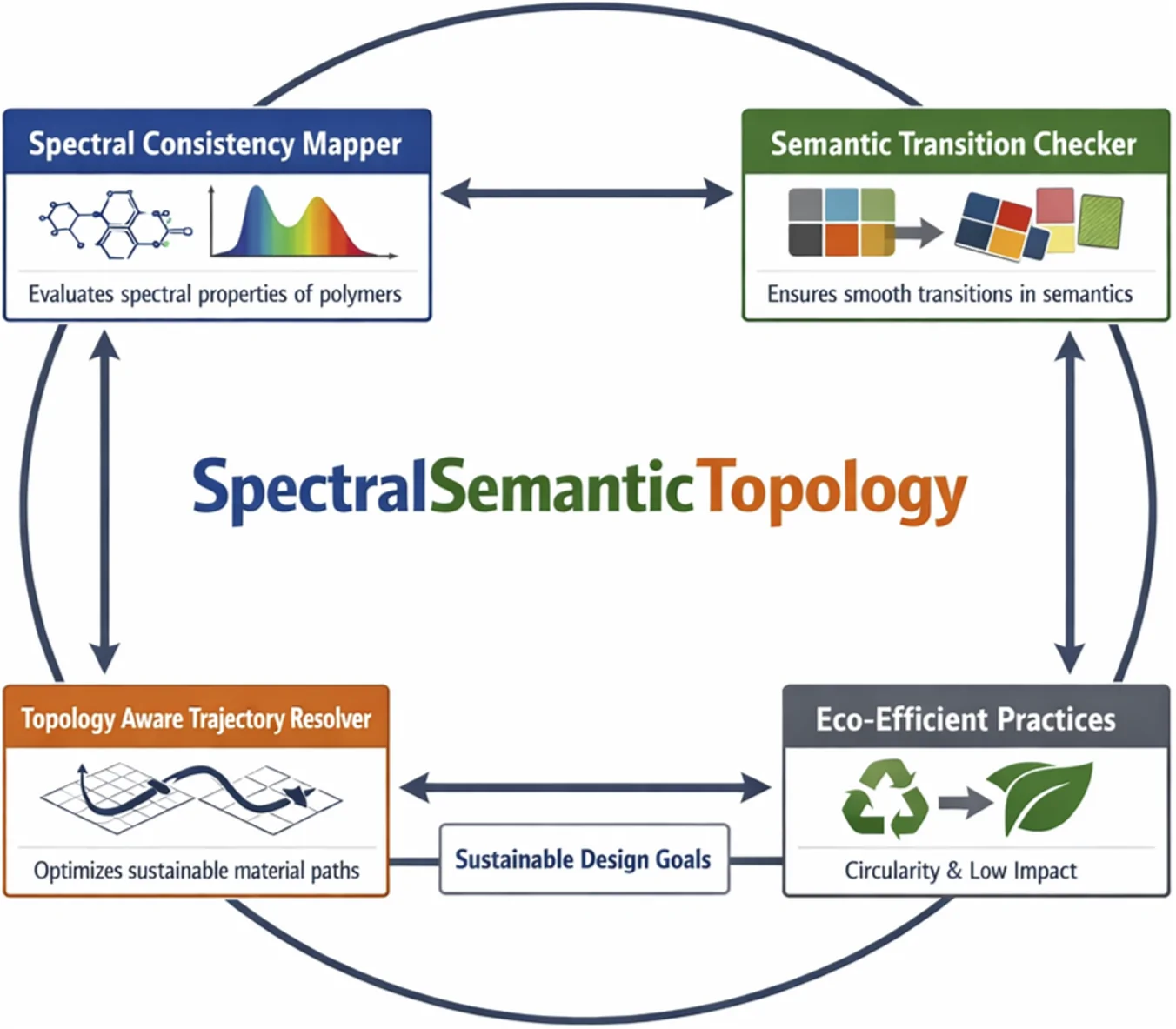
Diagram illustrating the SpectralSemanticTopology framework for sustainable jewelry design. The framework integrates four interconnected modules: Spectral Consistency Mapper, Semantic Transition Checker, Topology Aware Trajectory Resolver, and Eco Efficient Practices. These modules collectively optimize polymer spectral properties, semantic coherence, topological robustness, and environmental impact, ensuring alignment with sustainable design goals.

As shown in Figure 2, this subsection introduces the SpectralSemanticTopology framework for sustainable jewelry material design through green polymers and low impact processing strategies. The framework is formulated as a unified modeling and optimization process for three coupled design dimensions: environmental performance, perceptual attributes, and structural integrity. The Spectral Consistency Mapper models property related spectral behavior associated with visual appearance and optical response. The Semantic Transition Checker evaluates whether material substitution and processing transitions remain consistent with design intent, tactile quality, and sustainability requirements. The Topology Aware Trajectory Resolver captures structural regularity and process related feasibility in polymer molecular and material representations. These modules operate as coordinated components of a single framework, and their outputs are integrated through harmonic relation modeling and rule guided inference to support polymer property prediction, material candidate screening, and sustainability oriented optimization. The core methodological objective of SpectralSemanticTopology is to jointly optimize environmental performance, perceptual consistency, and structural regularity within one computational formulation. This design avoids the separate optimization of sustainability indicators, appearance related properties, and topology related constraints, because isolated optimization may produce material candidates that perform well on one criterion but fail under jewelry grade requirements. The unified objective therefore couples spectral fidelity, semantic consistency, and topological regularity so that polymer candidates can be evaluated according to both material performance and sustainable processing feasibility.

The AI contribution of the proposed framework is defined at four levels. It serves as a supervised polymer property prediction model that learns the mapping from polymer molecular structure representations to numerical material property values. This predictive function provides the measurable basis for ranking candidate polymers before experimental fabrication. It constructs coupled latent embedding spaces for spectral, semantic, and topological representations, allowing the model to encode appearance related responses, design level suitability, and molecular structural regularity within a shared learning process. It introduces a multi objective learning formulation in which the regression loss is jointly optimized with spectral consistency, semantic transition, and topology aware regularization terms. This enables the learned representation to consider property accuracy, perceptual consistency, structural feasibility, and sustainability related constraints simultaneously. It functions as a decision support framework for sustainable material screening, where predicted property values are combined with rule guided sustainability and processing constraints to filter candidate polymers. The technical contribution is not a general use of artificial intelligence, but a structure property prediction and screening framework that integrates embedding space modeling, multi objective representation learning, and constraint guided material decision support.

**FIGURE 3:**
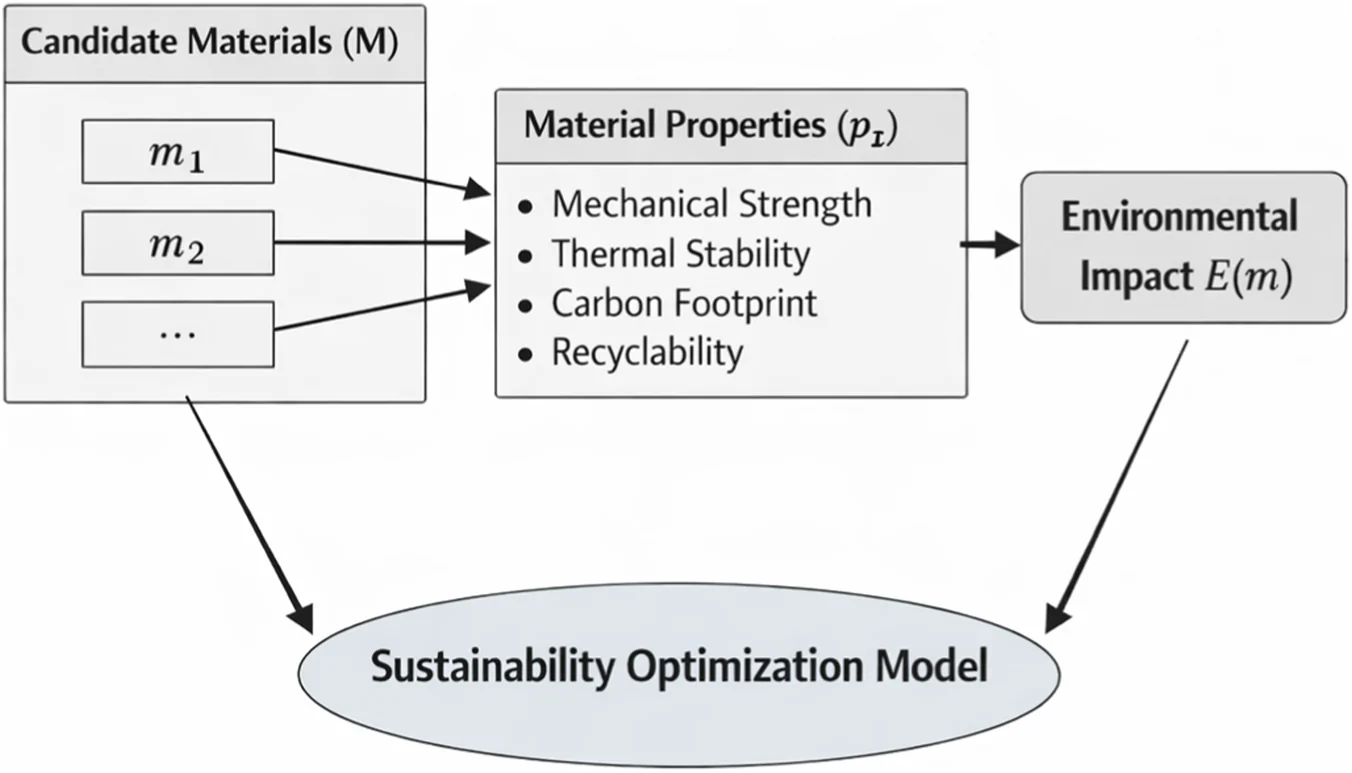
Diagram illustrating the sustainable jewelry design framework. Candidate materials (M) are evaluated based on their properties, including mechanical strength, thermal stability, carbon footprint, and recyclability. The environmental impact of each material (E(m)) is quantified and integrated into a sustainability optimization model to minimize overall impact while adhering to design constraints.

Spectral Consistency and Semantic Transition Integration: As shown in Figure 3, this component jointly models appearance related spectral consistency and design level semantic consistency within the SpectralSemanticTopology framework. The purpose of this component is not to treat spectral and semantic information as two independent innovations, but to connect material response and jewelry design suitability in a shared representation space. The Spectral Consistency Mapper ensures that the spectral properties of the materials align with sustainable design principles. This is achieved through a spectral analysis function S(λ) , where λ represents the wavelength of the spectral data. In implementation, $S\left(\lambda_{i};\theta \right)$ denotes the spectral or property related response predicted from the learned polymer representation at the sampled spectral index or property index $\lambda_{i}$ . The target profile $S_{\text{target}}\left(\lambda_{i}\right)$ is obtained from the normalized property related target representation or design requirement associated with the training sample. This formulation follows the general principle of representation alignment in materials informatics, where molecular structures are projected into property aligned latent spaces to improve material property prediction and interpretation (Butler et al., 2018; Ramakrishna et al., 2019). The optimization of spectral consistency is expressed as Equation 10:

$$\underset{\theta }{\min}\overset{N}{\underset{i=1}{\sum }}{\left(S\left(\lambda_{i};\theta \right)-S_{\text{target}}\left(\lambda_{i}\right)\right)}^{2}$$ (10)

Here, θ denotes the parameters of the spectral model, N is the number of spectral samples, and $S_{\text{target}}\left(\lambda_{i}\right)$ represents the target spectral profile for sustainable materials. The squared deviation term penalizes inconsistencies between the predicted response and the target response, thereby encouraging polymers with similar appearance related or performance related properties to be embedded close to each other in the spectral latent space. In the implemented model, the Spectral Consistency Mapper is realized as a multilayer neural projection that receives the encoded molecular representation and outputs the spectral latent vector. The resulting loss is used as an auxiliary constraint during supervised regression training.

Simultaneously, the Semantic Transition Checker ensures smooth transitions in the semantic properties of materials during processing. This is modeled using a semantic mapping function T(x) , where x is the semantic feature vector. The semantic feature vector x is derived from the fused molecular representation and encodes design suitability, tactile consistency, sustainability preference, and processing compatibility. The transition variable t∈[0,1] represents a continuous interpolation between the original design requirement and a greener material or processing configuration. In practical computation, the integral is approximated through discrete interpolation steps in the latent space. The transition is governed by Equation 11:

$$T\left(x\right)=\int_{0}^{1}\phi \left(x,t\right)\;dt$$ (11)

In this equation, ϕ(x,t) is a transition function that models the semantic change over time t . The function ϕ(x,t) is implemented as a gated consistency score generated by the Semantic Transition Checker. A higher inconsistency score indicates that the material substitution or processing change may alter design relevant attributes, such as perceived quality, surface feel, or jewelry use suitability. The semantic transition term is therefore used to regularize the learned representation so that sustainable material substitution does not rely only on numerical property improvement but also preserves design level compatibility. This idea is consistent with molecular representation learning and graph based prediction studies in which latent features are constrained to preserve application relevant information across related molecular configurations (Duvenaud et al., 2015; Gilmer et al., 2017). By integrating these two components, the model ensures that both spectral and semantic properties are optimized in tandem, providing a robust foundation for sustainable material design.

Topology Aware Trajectory Optimization: The second innovation focuses on the spatial and structural aspects of material design through the Topology Aware Trajectory Resolver. Within the implemented framework, topology refers to the molecular graph structure and learned structural regularity of a polymer candidate, rather than only to geometric coordinates in a physical object. This module employs a topology function τ(y) , where y represents the spatial coordinates. Here, y denotes the node level or graph level coordinate in the learned molecular representation, and τ(y) denotes the latent topological state associated with polymer connectivity and structural consistency. A topological trajectory represents the sequence of latent graph states updated during representation learning and candidate optimization. The trajectory resolution is achieved by solving the following differential equation (Equation 12):

$$\frac{d\tau \left(y\right)}{dt}=\nabla \cdot \left(\kappa \left(y\right)\nabla \tau \left(y\right)\right)$$ (12)

Here, κ(y) is the diffusion coefficient that governs the rate of topology change. In implementation, this diffusion style formulation is approximated by graph diffusion layers. Node features are updated according to molecular connectivity and learned edge weights, so that neighboring atoms or structural units exchange information in a controlled manner. The coefficient κ(y) is represented by learnable propagation weights that determine how strongly structural information is smoothed across the polymer graph. The purpose is not to simulate physical diffusion directly, but to regularize molecular representations and reduce structurally implausible latent states. Graph message passing and graph convolution provide the theoretical basis for using molecular connectivity to learn structure property relationships (Duvenaud et al., 2015; Gilmer et al., 2017).

The topology aware term contributes to sustainable manufacturing through structural feasibility and process efficiency. In polymer based jewelry fabrication, candidates with unstable molecular or geometric structures may lead to failed forming, excessive support material, high material loss, repeated processing, or premature failure during use. By regularizing the latent structural trajectory, the Topology Aware Trajectory Resolver favors polymer candidates with coherent graph level connectivity, smoother structural transitions, and reduced risk of process induced irregularity. This supports the selection of lightweight yet stable structures, reduces unnecessary reprocessing and material waste during early stage screening, and improves the likelihood that selected candidates can satisfy durability and manufacturability requirements under low impact processing conditions. Topology aware optimization is connected to sustainability not by directly estimating carbon emissions, but by improving the structural robustness and manufacturing compatibility of selected polymer candidates. This role is important because sustainable material selection requires candidates that are not only environmentally preferable, but also stable, durable, and efficient to fabricate. The integration of topology aware optimization into the framework allows for precise control over the spatial properties of the materials, ensuring that they meet the desired sustainability criteria.

Unified Optimization Framework for Sustainability: The third innovation lies in the integration of the previously described spectral, semantic, and topology aware modules into a unified optimization framework. This part serves as the overall training and optimization objective of the SpectralSemanticTopology framework. The three preceding components are therefore coordinated functional modules within one model rather than separate and unrelated contributions. This framework is designed to jointly optimize multiple dimensions of sustainable material design, ensuring that physical properties, semantic interpretability, and structural feasibility are coherently aligned within a single mathematical formulation.

The framework introduces a composite objective function that captures the interplay among spectral fidelity, semantic consistency, and topological regularity (Equation 13):

$$L=\alpha \overset{N}{\underset{i=1}{\sum }}{\left(S\left(\lambda_{i};\theta \right)-S_{\text{target}}\left(\lambda_{i}\right)\right)}^{2}+\beta \int_{0}^{1}\phi \left(x,t\right)\;dt+\gamma \int {\left(\nabla \cdot \left(\kappa \left(y\right)\nabla \tau \left(y\right)\right)\right)}^{2}\;dy$$ (13)

In this formulation, the first term represents the spectral reconstruction loss, where $S\left(\lambda_{i};\theta \right)$ denotes the predicted spectral response parameterized by θ , and $S_{\text{target}}\left(\lambda_{i}\right)$ corresponds to the desired spectral profile. This component ensures that the generated material configurations satisfy predefined optical or energy related constraints, which are critical in sustainable jewelry design where light interaction and material efficiency play essential roles. During training, this term is computed from the output of the Spectral Consistency Mapper and is used as an auxiliary alignment loss for the learned polymer representation.

The second term introduces a semantic regularization component through the function ϕ(x,t) , which encodes time dependent semantic consistency across the design space. By integrating over the temporal domain t∈[0,1] , the model captures the evolution of semantic attributes, such as aesthetic coherence, cultural symbolism, and user defined sustainability criteria. This term enables the framework to incorporate high level design intent and contextual meaning, bridging the gap between purely physical optimization and human centered design considerations. In the implemented workflow, the continuous transition is approximated by a finite number of latent interpolation steps, and the Semantic Transition Checker produces a consistency penalty for each step. The resulting semantic loss guides the model toward material candidates that remain compatible with jewelry design requirements while satisfying sustainability oriented constraints.

The third term enforces topological smoothness and structural integrity through a diffusion based operator. Here, κ(y) represents a spatially varying conductivity or material property field, while τ(y) denotes a latent structural function. The divergence term ∇⋅(κ(y)∇τ(y)) models the flow of structural information across the material domain. Squaring and integrating this quantity penalizes irregularities, discontinuities, and physically implausible configurations, thereby ensuring manufacturability and long term durability of the designed materials. In practice, this term is calculated from graph diffusion outputs and functions as a topological regularization loss. It constrains the learned molecular graph representation to preserve structural coherence during property prediction and candidate screening.

The weighting coefficients α , β , and γ serve as tunable hyperparameters that balance the relative importance of spectral accuracy, semantic alignment, and topological consistency. By adjusting these parameters, the framework can be adapted to different application scenarios, such as prioritizing environmental performance, aesthetic qualities, or structural robustness. These coefficients are selected according to validation performance, and the supervised regression loss remains the primary optimization target. The complete training objective can be written as Equation 14:

$$L_{\text{train}}=L_{\text{reg}}+\alpha L_{\text{spec}}+\beta L_{\text{sem}}+\gamma L_{\text{topo}},$$ (14)

where $L_{\text{reg}}$ is the mean squared error between the predicted polymer property value and the dataset label, $L_{\text{spec}}$ is the spectral consistency loss, $L_{\text{sem}}$ is the semantic transition loss, and $L_{\text{topo}}$ is the topology aware regularization loss. This training objective clarifies how the mathematical terms enter the actual learning process.

The unified nature of this optimization framework enables joint learning and co adaptation among the three components. Rather than optimizing each module independently, the model captures cross domain dependencies, allowing improvements in one aspect, such as spectral consistency, to inform and enhance others, such as semantic compatibility or graph level structural regularity. This holistic approach significantly improves convergence stability and solution quality in complex design spaces. The SpectralSemanticTopology model is inherently extensible. Additional modules such as lifecycle assessment metrics, carbon footprint estimators, or recyclability constraints can be seamlessly integrated into the objective function as new regularization terms. This modular extensibility ensures that the framework remains responsive to emerging sustainability standards and technological advancements.

The implemented procedure follows a clear computational workflow. Each polymer repeating unit is standardized into a canonical molecular structure. Descriptor based, graph based, and token based representations are constructed from the same polymer structure. These representations are projected into spectral, semantic, and topological latent spaces. The Spectral Consistency Mapper, Semantic Transition Checker, and Topology Aware Trajectory Resolver compute the corresponding auxiliary constraints. Harmonic relation modeling fuses the module outputs into a shared material representation. The regression head predicts the target polymer property value, and rule guided inference filters candidate materials according to environmental and low impact processing constraints. This procedure connects the mathematical formulation with the implemented training and screening pipeline, thereby improving reproducibility and practical interpretability.

### 3.4 Harmonic relation modeling and rule guided inference

**FIGURE 4:**
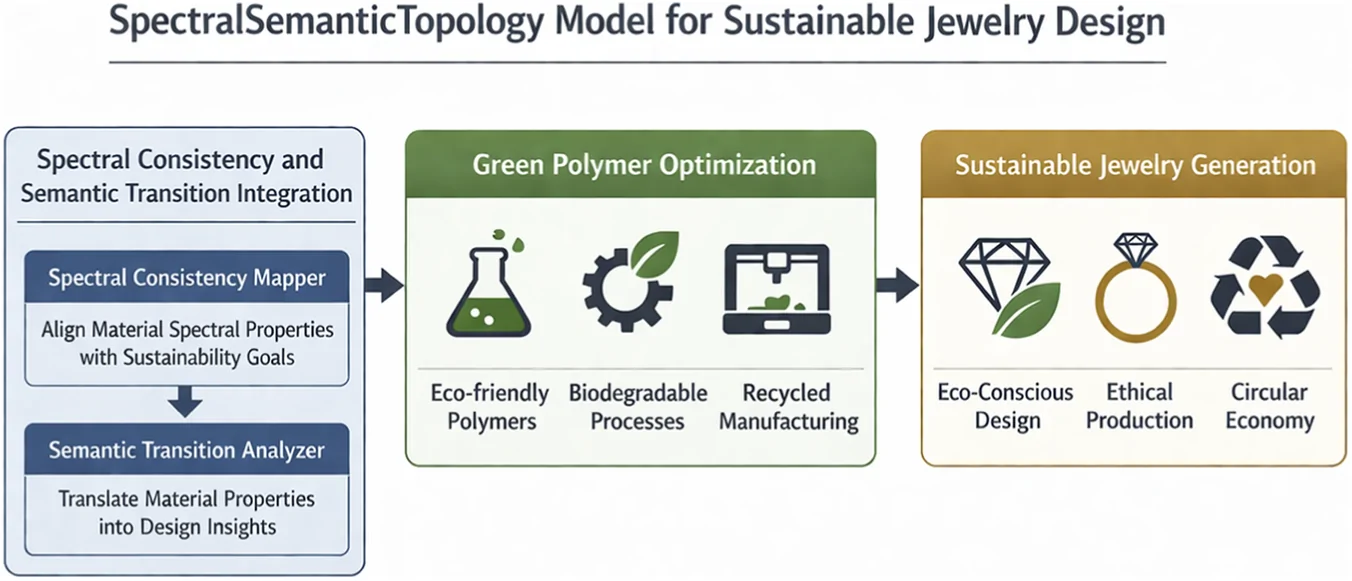
The SpectralSemanticTopology model integrates three modules to enhance sustainable jewelry design. The Spectral Consistency and Semantic Transition Integration module aligns material spectral properties with sustainability goals and translates semantic properties into design insights. The Green Polymer Optimization module focuses on eco friendly polymers, biodegradable processes, and recycled manufacturing. The Sustainable Jewelry Generation module ensures eco conscious design, ethical production, and circular economy principles.

As shown in Figure 4, In the pursuit of sustainable design for jewelry materials, the integration of green polymers and low impact processing strategies necessitates a sophisticated approach to model the intricate relationships between material properties and environmental impact. Our strategy, termed Harmonic Relation Modeling and Rule Guided Inference, is designed to address these challenges by leveraging the SpectralSemanticTopology framework. This strategy is pivotal in ensuring that the design process not only meets aesthetic and functional requirements but also adheres to sustainability principles.

**FIGURE 5:**
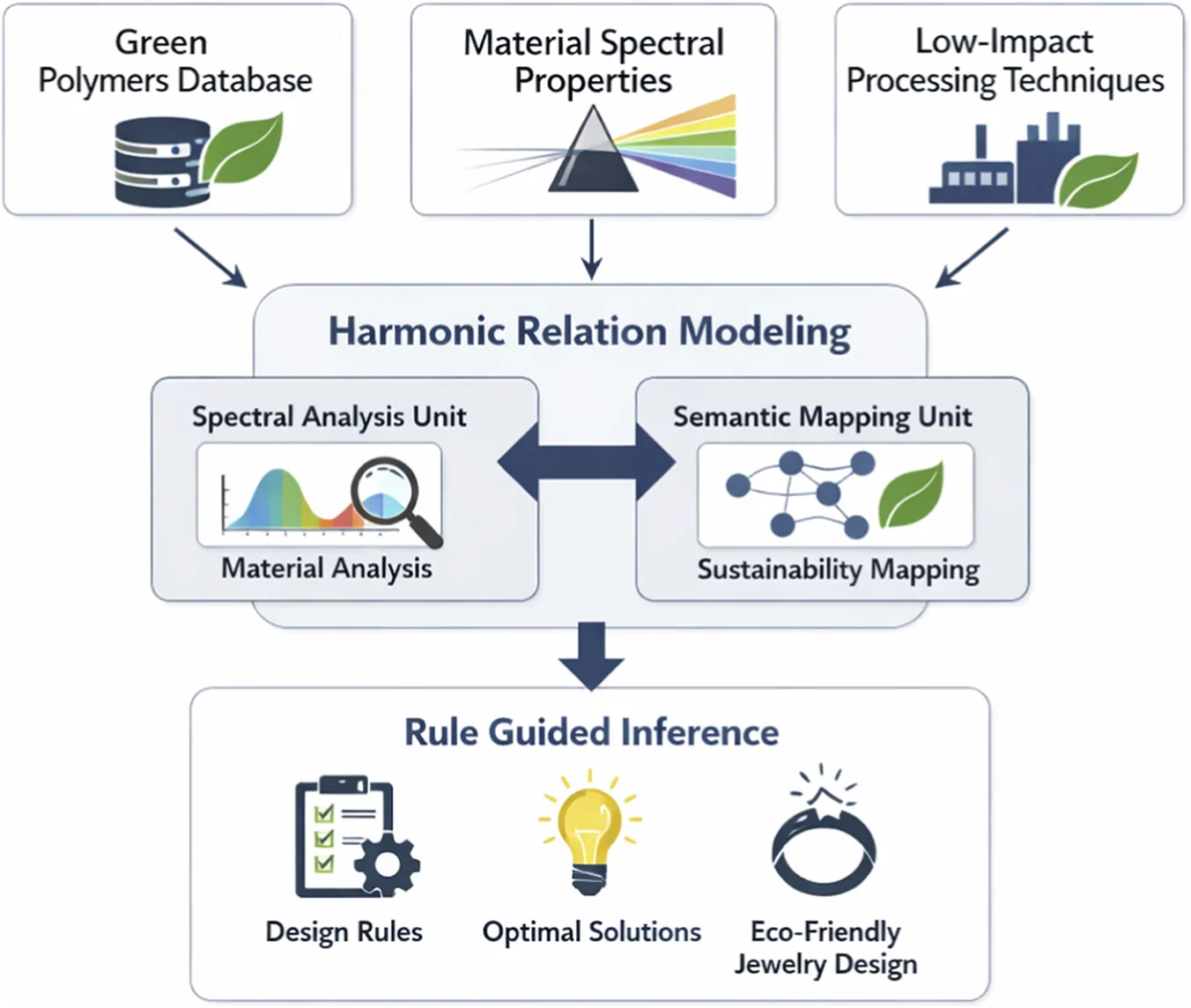
The Sustainable Jewelry Design Framework integrates green polymers, material spectral properties, and low impact processing techniques to achieve eco friendly jewelry design. Harmonic Relation Modeling establishes connections between spectral data and sustainability metrics, while Rule Guided Inference ensures adherence to design rules and environmental objectives. This approach facilitates optimal solutions that balance aesthetic, functional, and ecological considerations.

Harmonic Relation Modeling: As shown in Figure 5, This component focuses on establishing a coherent relationship between the spectral properties of materials and their semantic implications in sustainable design. By utilizing the Spectral Consistency Mapper, we can accurately capture the spectral characteristics of green polymers, which are essential for understanding their environmental footprint. This module allows us to map these properties onto a semantic space that reflects the sustainability goals of the design process. The mapping is achieved through a series of transformations that preserve the integrity of the spectral data while aligning it with the semantic objectives.

Let S represent the spectral properties of a material, and M(S) denote the mapping function that transforms S into a semantic space H . The mapping function is defined as Equation 15:

$$M\left(S\right)=\int_{\Omega }f\left(S,\lambda \right)\;d\lambda$$ (15)

where Ω is the spectral domain, λ is the wavelength, and f(S,λ) is a weighting function that aligns spectral data with semantic objectives. The semantic space H is structured to encapsulate sustainability metrics, such as carbon footprint and recyclability, ensuring that the mapped data is both meaningful and actionable.

The Spectral Consistency Mapper ensures that the mapping process adheres to the principle of spectral consistency, which is mathematically expressed as Equation 16:

$$\|M\left(S_{1}\right)-M\left(S_{2}\right)\|\leq \epsilon$$ (16)

for any two materials $S_{1}$ and $S_{2}$ with similar sustainability profiles, where ϵ is a predefined tolerance threshold. This guarantees that materials with comparable environmental impacts are represented similarly in the semantic space.

Rule Guided Inference: This component complements the modeling process by providing a structured approach to decision making in the design process. This involves the Semantic Transition Checker, which ensures that transitions between different design states adhere to predefined sustainability rules. These rules are derived from a comprehensive analysis of environmental impact factors and are encoded into the system to guide the inference process. The checker operates by evaluating potential design transitions against these rules, ensuring that each step in the design process contributes positively to the overall sustainability of the jewelry materials.

Let T represent a design transition, and R denote the set of sustainability rules. The Semantic Transition Checker evaluates T using the function C(T,R) , defined as Equation 17:

$$C\left(T,R\right)=\left\{\begin{array}{cc} 1 & \text{if }T\text{ satisfies all }R\\ 0 & \text{otherwise} \end{array}\right.$$ (17)

A transition T is only accepted if C(T,R)=1 , ensuring strict adherence to sustainability principles.

The rules R are encoded as a set of constraints, such as Equation 18:

$$g_{i}\left(T\right)\leq 0,\;i=1,2,\ldots ,n$$ (18)

where $g_{i}$ represents individual sustainability criteria, and n is the total number of criteria. These constraints are designed to enforce environmentally responsible practices throughout the design process.

Topology Aware Trajectory Resolution: This module plays a crucial role in harmonizing the design trajectory with sustainability objectives. It is responsible for resolving the complex trajectories that arise during the design process, ensuring that they are topology aware and aligned with the harmonic relations established earlier. By doing so, it facilitates a seamless integration of design elements that are both aesthetically pleasing and environmentally responsible.

Let P represent a design trajectory, and $T_{\text{opt}}$ denote the optimized trajectory. The Topology Aware Trajectory Resolver computes $T_{\text{opt}}$ by solving the optimization problem (Equation 19):

$$T_{\text{opt}}=\arg\underset{P}{\min}\int_{P}h\left(x\right)\;dx$$ (19)

where h(x) is a cost function that quantifies the deviation from sustainability objectives at each point x along the trajectory P .

The trajectory P is subject to topological constraints, expressed as Equation 20:

$$\phi_{j}\left(P\right)=0,\;j=1,2,\ldots ,m$$ (20)

where $\phi_{j}$ represents individual topological requirements, and m is the total number of constraints. These constraints ensure that the trajectory remains consistent with the harmonic relations established in the modeling phase.

The synergy between Harmonic Relation Modeling and Rule Guided Inference is what sets our strategy apart. It allows for a dynamic and responsive design process that can adapt to changing sustainability requirements while maintaining the integrity of the design vision. This strategy not only enhances the capability of the SpectralSemanticTopology framework but also provides a robust foundation for future advancements in sustainable jewelry design.

### 3.5 Method workflow

To clarify the practical implementation of the proposed framework, the complete methodological flow is organized into four stages: input data, model architecture, training and adjustment, and output results. This structured description specifies how system specific variables enter the framework, how they are processed, and what measurable outputs are generated.

Input data. The input of the framework is a polymer candidate represented by its molecular structure. Each polymer structure is standardized into a canonical molecular representation before model processing. Three types of computable inputs are constructed from the same polymer candidate. The first type is a descriptor based vector, which contains molecular and material related features such as atom composition, bond information, structural descriptors, and available property indicators. The second type is a graph based representation, in which atoms are treated as nodes and chemical bonds are treated as edges. Node features include atom type, valence, aromaticity, formal charge, and hybridization, while edge features include bond type and conjugation state. The third type is a token based representation, in which the canonical polymer string is converted into a sequence of tokens for structure level encoding. In addition to molecular information, sustainability and processing related variables can be introduced when available, including carbon footprint, recyclability, processing temperature, curing time, energy demand, material loss rate, and compatibility with low impact processing. These variables provide the basis for environmental evaluation and rule guided filtering.

Model architecture. The model architecture follows the SpectralSemanticTopology framework. The descriptor based input, graph based input, and token based input are first encoded into latent molecular representations. These representations are then projected into three coupled latent spaces. The spectral latent space captures property related and appearance related responses associated with optical behavior, color stability, and surface performance. The semantic latent space captures design suitability, tactile consistency, sustainability preference, and compatibility with jewelry use. The topological latent space captures molecular connectivity, graph level structural regularity, and process related feasibility. The Spectral Consistency Mapper processes the spectral latent representation, the Semantic Transition Checker processes the semantic latent representation, and the Topology Aware Trajectory Resolver processes the topological latent representation. The outputs of these modules are integrated through harmonic relation modeling, while rule guided inference imposes sustainability and processing constraints. The fused representation is finally passed to a regression head to predict the target polymer property value.

Training and adjustment. The framework is trained under a supervised regression setting. Each training sample consists of a polymer molecular structure and its recorded numerical property value. During training, the model predicts the property value $\hat{y}$ , and the primary regression loss is computed between $\hat{y}$ and the dataset label y . In addition to the regression loss, auxiliary consistency terms are used to guide representation learning. The spectral consistency term encourages the predicted spectral or property related response to match the target profile. The semantic transition term penalizes latent transitions that violate design suitability or sustainability consistency. The topological regularization term constrains the molecular graph representation to preserve structural coherence. The overall training objective is expressed as Equation 21:

$$L_{\text{train}}=L_{\text{reg}}+\alpha L_{\text{spec}}+\beta L_{\text{sem}}+\gamma L_{\text{topo}},$$ (21)

where $L_{\text{reg}}$ is the supervised regression loss, $L_{\text{spec}}$ is the spectral consistency loss, $L_{\text{sem}}$ is the semantic transition loss, and $L_{\text{topo}}$ is the topology aware regularization loss. The weights α , β , and γ are selected according to validation performance. Model parameters are updated by gradient based optimization, and the final model is selected according to validation error before evaluation on the held out test set.

Output results. The main output of the framework is the predicted polymer property value $\hat{y}$ , which is a measurable numerical indicator used to evaluate polymer material suitability. This value supports early stage screening of green polymer candidates before fabrication and processing. In addition to the predicted property value, the framework generates intermediate latent representations in the spectral, semantic, and topological spaces. These representations provide interpretable information about appearance related consistency, design compatibility, and structural regularity. During material screening, the predicted property value is combined with environmental and processing constraints to produce a ranked and filtered candidate list. The final output therefore includes measurable regression results, such as MAE, RMSE, $R^{2}$ , and Pearson correlation during evaluation, as well as a selected subset of polymer candidates that satisfy property performance, sustainability requirements, and low impact processing feasibility.

## 4 Experiments

### 4.1 Task definition

The experimental task is defined as polymer property prediction for sustainable jewelry material design. In this study, property prediction is used as an early-stage screening step for identifying green polymer candidates before fabrication and processing. This setting is based on the fact that polymer molecular structures determine key material properties, and these properties affect whether a polymer can satisfy jewelry-grade requirements such as stability, durability, processability, appearance-related performance, and compatibility with low impact processing. Accurate prediction can therefore help prioritize promising polymers for further evaluation and exclude unsuitable candidates at an early stage, reducing unnecessary material trials and processing cost. The input X is the molecular structure representation of a polymer, encoded from its repeating unit and converted into a computable molecular descriptor, molecular graph, or tokenized structure sequence for model training. Each input instance corresponds to one polymer candidate that may serve as a potential material for sustainable jewelry applications. The output Y is the numerical target property value of the polymer, representing a quantitative material performance indicator used to evaluate green polymer candidates. The learning paradigm is supervised regression. The model learns a mapping from polymer molecular structure representation to polymer target property value, so that the predicted value can support material screening, sustainable design evaluation, and the selection of polymer candidates with desirable performance. The label for each training instance is the target property value recorded in the polymer property datasets. Supervision is obtained from system statistics in the Polymer Genome Dataset and the PI1M Dataset. The supervision signal is not generated from manual category annotation, implicit user behavior, or expert trajectories, but from curated polymer property statistics associated with each molecular structure. During training, each polymer molecular structure representation is paired with its recorded numerical property value, and the regression model is optimized to reduce the difference between the predicted value and the dataset label. This setting provides a measurable basis for comparing the proposed method with Random Forest, XGBoost, MLP, GCN, ChemBERTa, and LightGBM.

### 4.2 Dataset and data preprocessing

#### 4.2.1 Datasets

The experiments use two public polymer informatics datasets. The Polymer Genome Dataset is used as the primary supervised regression dataset for small scale high quality polymer property prediction. It contains 1073 polymer and polymer related material records, each represented by a molecular structure and associated numerical target properties. This public dataset is selected because it provides curated polymer structure property records for evaluating supervised structure property learning. In the present task, the recorded numerical property is used as a measurable proxy for early stage polymer suitability assessment. In this study, the target property is treated as a quantitative indicator of polymer material suitability, because it reflects a property dimension related to the feasibility of using the polymer in jewelry grade applications. Jewelry grade suitability is operationalized as a downstream screening objective involving stability, durability, processability, appearance related performance, and compatibility with low impact processing, rather than as a predefined category label in the original data source. Samples are retained only when the molecular structure is valid, the polymer repeating unit is parsable, and the target property value is available. Duplicated molecular structures are removed after canonical structure normalization, and records with missing property values are discarded. The dataset is randomly divided into training, validation, and test subsets with an 8:1:1 ratio, and the same split protocol is repeated in ten independent runs with different random seeds. The PI1M Dataset is used as the large scale polymer structure dataset for representation learning and transfer validation. It contains approximately 1,000,000 polymer structures generated for polymer informatics benchmarking. This public dataset is selected because it provides a broad polymer structure space for evaluating representation learning and large scale candidate exploration. For sustainable jewelry material screening, PI1M provides a broader polymer structure space for testing whether the proposed method can support large scale candidate exploration. In this study, PI1M is used as a candidate polymer structure source, while jewelry related requirements are incorporated through downstream prediction, constraint filtering, and material screening. Samples are retained when the polymer structure string is valid, the molecular graph is successfully constructed, and the corresponding target value is present for the evaluated regression task. Invalid structures, duplicated canonical structures, and records without target values are removed. PI1M is randomly split into training, validation, and test subsets with an 8:1:1 ratio, using structure level isolation so that the same canonical polymer structure appears in only one subset.

Table 1 summarizes the source, availability, scale, variables, labels, selection reasons, preprocessing steps, and access links of the datasets. The experiments are based on public polymer informatics resources, and these resources provide molecular structures and numerical property labels for supervised polymer property prediction. The sustainable jewelry material design task is constructed by using these property labels for early stage prediction and by applying downstream constraints related to stability, durability, processability, appearance related performance, and compatibility with low impact processing.

**TABLE 1:** Summary of datasets used for polymer property prediction and sustainable jewelry material screening.

Dataset	Source	Public	Sample size	Variables	Task label	Selection reason	Preprocessing	Access link
Polymer genome dataset	Polymer genome platform and related polymer informatics literature	Yes	1073 records after task selection	Polymer molecular structure, molecular descriptors, and numerical polymer property values	Numerical polymer property value for supervised regression	Small-scale curated polymer property prediction benchmark for evaluating structure–property learning ability	Invalid structures, unparsable repeating units, duplicated canonical structures, and records without target values are removed. Descriptor values and target labels are normalized using training subset statistics	https://khazana.gatech.edu/dataset/
PI1M dataset	PI1M benchmark database for polymer informatics	Yes	Approximately 1,000,000 polymer structures before filtering	Polymer structure strings, molecular graph representations, tokenized structure sequences, and available task-specific property values	Numerical polymer property value for supervised regression or transfer validation	Large-scale polymer structure space for representation learning, transfer validation, and candidate screening	Invalid structure strings, failed graph construction records, duplicated canonical structures, and records without target values are removed. Structure-level isolation is applied before splitting	https://github.com/RUIMINMA1996/PI1M

#### 4.2.2 Data preprocessing

All polymer records from the Polymer Genome Dataset Kim et al. (2023) and the PI1M Dataset Ma and Luo (2020) are processed with the same structure centered pipeline before model training. The purpose of this pipeline is to align heterogeneous polymer records into a unified representation format so that descriptor based, graph based, and token based inputs describe the same polymer candidate. Data cleaning first removes duplicated polymers by converting each molecular structure into a canonical structure string and retaining one record for each canonical polymer. Records with missing molecular structures, failed parsing results, or missing target property values are discarded. Abnormal samples are removed when the numerical target property is outside the range defined by the first quartile minus three interquartile ranges and the third quartile plus three interquartile ranges within the training subset. This rule is applied only on the training statistics, and the validation and test subsets are filtered by the thresholds derived from the training subset to avoid information leakage.

Structure standardization is then performed by converting each polymer repeating unit into a canonical SMILES representation with explicit polymer connection atoms retained. For descriptor based models, the canonical structure is transformed into a fixed length molecular descriptor vector, and each descriptor dimension is normalized by z score scaling using the mean and standard deviation computed from the training subset. For graph based models, atoms are mapped to node features including atom type, valence, aromaticity, formal charge, and hybridization, while chemical bonds are mapped to edge features including bond type and conjugation state. For ChemBERTa, each canonical polymer string is tokenized with the pretrained tokenizer, truncated to 256 tokens, and padded to 256 tokens with an attention mask. For the proposed framework, the three representation types are generated from the same canonical polymer structure and then projected into spectral, semantic, and topological latent spaces. This alignment strategy ensures that the model integrates different views of the same material candidate rather than combining unrelated heterogeneous records. Target property values are standardized by z score normalization during training and transformed back to the original scale for MAE, RMSE, $R^{2}$ , and Pearson Correlation calculation. The prediction label is the numerical polymer property value provided by the corresponding dataset. Sustainability and processing related variables are used as downstream screening constraints and rule guided filtering criteria when corresponding information is available. This label construction strategy keeps the supervised learning target consistent with measured or recorded property values while allowing sustainability oriented requirements to guide candidate selection after prediction. No sequence windowing, frame sampling, image augmentation, speech augmentation, or external visual feature extraction is used, because the task input is a polymer molecular structure representation rather than temporal, visual, or acoustic data.

### 4.3 Evaluation metrics and baseline

#### 4.3.1 Metrics definition

The evaluation uses four primary regression metrics and two efficiency metrics. Mean Absolute Error measures the average absolute deviation between the predicted polymer property value and the ground truth value, and it is defined as $MAE=\frac{1}{n}\sum_{i=1}^{n}|{\hat{y}}_{i}-y_{i}|$ . Root Mean Square Error measures the square root of the mean squared prediction error, and it is defined as $RMSE=\sqrt{\frac{1}{n}\sum_{i=1}^{n}{\left({\hat{y}}_{i}-y_{i}\right)}^{2}}$ . The coefficient of determination evaluates the proportion of target variance explained by the model, and it is defined as $R^{2}=1-\frac{\sum_{i=1}^{n}{\left(y_{i}-{\hat{y}}_{i}\right)}^{2}}{\sum_{i=1}^{n}{\left(y_{i}-\bar{y}\right)}^{2}}$ . Pearson Correlation measures the linear agreement between predicted and ground truth property values, and it is computed between $\hat{y}$ and y . Lower MAE and RMSE indicate higher prediction accuracy, while higher $R^{2}$ and Pearson Correlation indicate stronger property prediction consistency. Efficiency is evaluated by Params and FLOPs. Params reports the number of trainable model parameters. FLOPs reports the number of floating point operations required for one forward inference pass on one polymer structure representation.

#### 4.3.2 Evaluation protocol

The evaluation protocol follows the supervised regression setting for polymer property prediction. Each polymer molecular structure representation is used as the input, and the corresponding numerical polymer property value is used as the target. The Polymer Genome Dataset is evaluated as the primary high quality property prediction benchmark, while the PI1M Dataset is used for large scale structure representation evaluation and transfer validation. All models are trained on the training split, selected according to validation MAE, and evaluated once on the held out test split for each random seed. The dataset split follows an 8:1:1 random structure level partition, where duplicated canonical polymer structures are removed before splitting. Structure level isolation is enforced so that the same canonical polymer does not appear in more than one subset. The setting is offline evaluation because all predictions are produced on fixed test sets after training is completed. Top K ranking, IoU thresholding, PCKh thresholding, time based splitting, user isolation, city isolation, and online feedback evaluation are not used, because the task is numerical polymer property regression rather than ranking, detection, pose estimation, temporal prediction, recommendation, or deployment interaction.

#### 4.3.3 Statistical settings

All experiments are repeated with N=10 independent runs using ten different random seeds. For each run, the model initialization, data split seed, and mini batch order are fixed by the corresponding seed to ensure reproducibility. The final results are reported as mean ± standard deviation over the ten independent runs. MAE, RMSE, $R^{2}$ , Pearson Correlation, Params, and FLOPs are reported under the same statistical protocol. Model comparison is performed between the proposed method and each baseline on the paired results from the ten runs. Statistical significance is tested using a two sided paired t test with p<0.05 . A performance difference is considered statistically significant only when the paired test satisfies this threshold. Validation results are used only for model selection and hyperparameter selection, while test results are used only for final reporting. The same preprocessing pipeline, data split ratio, target normalization rule, and metric computation procedure are applied to all compared models to ensure that the reported differences reflect model performance rather than evaluation inconsistency.

#### 4.3.4 Baseline

Six baseline models are used for comparison. Random Forest is included as the traditional machine learning baseline because it provides a strong nonparametric regression model for descriptor based polymer property prediction. XGBoost is included as an ensemble learning baseline because gradient boosted decision trees are widely used for structured molecular descriptors and often perform strongly on tabular materials data. MLP is included as the feedforward neural network baseline, using fixed length molecular descriptor vectors as input and numerical property values as regression targets. GCN is included as the graph neural network baseline because it directly operates on molecular graph structures and captures atom bond connectivity during message passing. ChemBERTa is included as the molecular pretrained model baseline, using tokenized polymer structure strings as input and a regression head for target property prediction. LightGBM is included as the lightweight gradient boosting baseline because it provides efficient training and inference with a small computational footprint. These baselines cover traditional regression, ensemble learning, neural regression, graph based deep learning, pretrained molecular modeling, and lightweight prediction.

### 4.4 Implementation details

All experiments are conducted on a workstation equipped with an Intel Xeon Gold 6230 CPU, one NVIDIA RTX 3090 GPU with 24 GB memory, 128 GB RAM, and Ubuntu 22.04 LTS. The software environment uses Python 3.10, PyTorch 2.1.0, CUDA 11.8, cuDNN 8.9, RDKit 2023.09, scikit learn 1.3.2, XGBoost 2.0.3, LightGBM 4.1.0, NumPy 1.26.2, and SciPy 1.11.4. Polymer molecular structures are parsed and standardized with RDKit before descriptor construction, molecular graph construction, and tokenizer based sequence encoding. PyTorch is adopted because it supports the joint optimization of descriptor based, graph based, token based, spectral, semantic, and topological modules within one computational graph. The main training settings are summarized in Table 2. AdamW is used to stabilize the optimization of heterogeneous molecular representations and to reduce overfitting through weight decay. Cosine annealing is used to support fast learning in early epochs and gradual refinement of spectral consistency, semantic compatibility, and topological regularity in later epochs. No image, speech, or temporal data augmentation is used, because the input is a polymer molecular structure rather than visual, acoustic, or time series data. Instead, robustness is supported by canonical structure standardization, descriptor normalization, dropout, weight decay, gradient clipping, and repeated random seed evaluation.

**TABLE 2:** Training settings of the proposed framework.

Setting	Value	Purpose
Optimizer	AdamW	Stable optimization with weight decay
Learning rate	$1\times 10^{-3}$	Initial model learning
Minimum learning rate	$1\times 10^{-6}$	Late stage refinement
Schedule	Cosine annealing	Smooth learning rate decay
Batch size	64	Balance stability and memory cost
Epochs	200	Sufficient regression training
Early stopping	30 epochs	Avoid overfitting
Weight decay	$1\times 10^{-5}$	Parameter regularization
Gradient clipping	5.0	Training stabilization
Dropout	0.1	Hidden feature regularization
Random seeds	0, 1, 2, 3, 4, 5, 6, 7, 8, 9	Ten run statistical reporting

All neural models are trained for 200 epochs with a batch size of 64, an initial learning rate of $1\times 10^{-3}$ , the AdamW optimizer, and a weight decay of $1\times 10^{-5}$ . The learning rate is controlled by cosine annealing with a minimum learning rate of $1\times 10^{-6}$ . Early stopping is applied when validation MAE does not improve for 30 consecutive epochs. Target property values are normalized with training set statistics during optimization and restored to the original scale during metric calculation. Gradient clipping is set to 5.0 to stabilize training. The primary loss function is mean squared error, and the checkpoint with the lowest validation MAE is selected for final test evaluation. The proposed model uses a molecular structure encoder followed by the SpectralSemanticTopology prediction module. The molecular descriptor branch uses a 512 dimensional input vector generated from canonical polymer structures and projects it into a 256 dimensional hidden representation. The graph branch represents each polymer as an atom bond graph with atom type, valence, aromaticity, formal charge, and hybridization as node features, and bond type and conjugation state as edge features. The graph encoder contains four message passing layers with a hidden dimension of 256 and residual connections between adjacent layers. The structure string branch uses a tokenizer length of 256 tokens and a 256 dimensional token embedding. The Spectral Consistency Mapper is implemented as a three layer multilayer perceptron with hidden dimensions of 256, 128, and 64. The Semantic Transition Checker is implemented as a two layer multilayer perceptron with a hidden dimension of 128 and sigmoid gated consistency output. The Topology Aware Trajectory Resolver uses two graph diffusion layers with a hidden dimension of 128 to model structural regularity. The three module outputs are fused by harmonic relation modeling through a 256 dimensional fusion layer. The final regression head contains two fully connected layers with hidden dimensions of 128 and 64, followed by a single scalar output for the predicted polymer property value. Dropout is set to 0.1 in hidden layers, and ReLU is used as the activation function. The training procedure follows a supervised regression workflow. Each polymer structure is first standardized into a canonical molecular representation. Descriptor based, graph based, and token based inputs are then generated from the same polymer candidate and projected into spectral, semantic, and topological latent spaces. The regression head predicts the numerical polymer property value, and the model is optimized by $L_{\text{train}}=L_{\text{reg}}+\alpha L_{\text{spec}}+\beta L_{\text{sem}}+\gamma L_{\text{topo}}$ , where $L_{\text{reg}}$ is the mean squared error between the predicted value and the dataset label, while $L_{\text{spec}}$ , $L_{\text{sem}}$ , and $L_{\text{topo}}$ guide spectral consistency, semantic compatibility, and topological regularity. This objective links the training setup with sustainable jewelry material design by improving property prediction while preserving representation feasibility for downstream candidate screening.

All baseline methods are trained and evaluated under the same data partitions, preprocessing rules, target normalization strategy, random seeds, hardware environment, and metric definitions as the proposed model. Hyperparameters for Random Forest, XGBoost, MLP, GCN, ChemBERTa, and LightGBM are selected using the validation set only. The test set remains unseen during model selection, and the final comparison is reported using the same ten run statistical protocol. Table 3 summarizes the computational implementation of the proposed framework. The input is a polymer molecular structure, which is transformed into descriptor based, graph based, and token based representations. These representations are encoded and projected into three coupled latent spaces. The spectral space describes appearance related and property related responses, the semantic space describes design suitability and sustainability preference, and the topological space describes molecular connectivity and structural regularity. The framework is trained as a supervised regression model. The primary prediction target is the numerical polymer property value recorded in the dataset. The regression loss is combined with auxiliary spectral, semantic, and topological losses, so that the learned representation considers prediction accuracy, perceptual consistency, design compatibility, and structural feasibility at the same time. During inference, the predicted property value is used for candidate ranking, and rule guided constraints are further applied to obtain material recommendations that satisfy sustainability and low impact processing requirements.

**TABLE 3:** Compact architecture specification of the proposed SpectralSemanticTopology framework.

Item	Specification
Input representation	Each polymer candidate is represented by a canonical molecular structure and encoded as descriptor based, graph based, and token based molecular representations
Latent space encoding	The encoded molecular representations are projected into spectral, semantic, and topological latent spaces, corresponding to appearance related response, design suitability, and molecular structural regularity
Model architecture	The framework consists of a molecular structure encoder, the spectral consistency mapper, the semantic transition checker, the topology aware trajectory resolver, harmonic relation modeling, rule guided inference, and a regression head
Learning objective	The training objective is defined as $L_{\text{train}}=L_{\text{reg}}+\alpha L_{\text{spec}}+\beta L_{\text{sem}}+\gamma L_{\text{topo}}$ , where the regression loss is combined with spectral consistency, semantic transition, and topology aware regularization terms
Optimization process	Model parameters are learned by gradient based optimization. Validation error is used for model selection, and the checkpoint with the lowest validation MAE is used for final evaluation
Output and recommendation	The direct output is the predicted numerical polymer property value. Candidate polymers are then ranked and filtered by environmental, structural, and processing constraints to generate sustainability oriented design recommendations

### 4.5 Results and discussion

#### 4.5.1 Comparative experiments

The comparative experiments evaluate the proposed method against six baselines specified in the experimental design: Random Forest, XGBoost, MLP, GCN, ChemBERTa, and LightGBM. The comparison is conducted on the Polymer Genome Dataset and the PI1M Dataset. The primary evaluation metrics are MAE, RMSE, $R^{2}$ , and Pearson Correlation, while Params and FLOPs are used to evaluate computational efficiency. The experiment is designed to test whether the proposed SpectralSemanticTopology based regression model improves polymer property prediction from molecular structure representations under the same data and supervision setting. All methods use identical preprocessing rules, identical training, validation, and test partitions, and identical target normalization procedures. Hyperparameters are selected only on the validation split, and the test split is used only for final reporting. Each experiment is repeated over ten independent random seeds, and the results are reported as mean ± standard deviation. Statistical significance is tested with a two sided paired t test at p<0.05 , ensuring fair and reproducible comparison across all methods.

Table 4 reports the main regression performance on the Polymer Genome Dataset, which is used as the high quality benchmark for polymer property prediction from molecular structure representations. The proposed method achieves the best results across all four evaluation metrics, with an MAE of 0.244±0.010 , an RMSE of 0.312±0.013 , an $R^{2}$ of 0.858±0.014 , and a Pearson Correlation of 0.928±0.009 . Compared with ChemBERTa, which is the strongest baseline in this table, the proposed method reduces MAE from 0.278 to 0.244, corresponding to a relative improvement of 12.23% , and reduces RMSE from 0.354 to 0.312, corresponding to a relative improvement of 11.86% . Meanwhile, $R^{2}$ increases from 0.821 to 0.858, and Pearson Correlation increases from 0.909 to 0.928, indicating that the proposed model not only lowers prediction error but also produces property estimates that are more consistent with the recorded polymer statistics. The improvement over descriptor based models such as Random Forest, XGBoost, and LightGBM suggests that fixed molecular descriptors alone are insufficient to fully capture the structure-property relationships required by sustainable jewelry material design. The gain over GCN further shows that ordinary atom-bond message passing benefits from additional sustainability-aware constraints. These results can be attributed to the proposed SpectralSemanticTopology framework, where the Spectral Consistency Mapper strengthens the alignment between polymer structure and property-related spectral responses, the Semantic Transition Checker preserves design-relevant semantic coherence during representation learning, and the Topology Aware Trajectory Resolver models structural regularity in the molecular graph. In addition, harmonic relation modeling enables the three representation spaces to be fused in a coordinated manner rather than optimized independently. The smaller standard deviations of the proposed method also indicate more stable behavior across the ten independent runs, supporting the robustness of the proposed sustainability-aware regression design.

**TABLE 4:** Main regression results on the Polymer Genome Dataset.

Method	MAE ↓	RMSE ↓	$R^{2}$ ↑	Pearson ↑
Random forest Munde and Kaur (2024)	0.342±0.018	0.431±0.021	0.728±0.024	0.856±0.016
XGBoost Huang et al. (2023)	0.305±0.015	0.389±0.018	0.781±0.020	0.886±0.013
MLP Liang et al. (2025)	0.326±0.019	0.407±0.022	0.756±0.026	0.872±0.017
GCN Park et al. (2022)	0.286±0.014	0.365±0.017	0.807±0.018	0.901±0.012
ChemBERTa Rollins et al. (2024)	0.278±0.013	0.354±0.016	0.821±0.017	0.909±0.011
LightGBM Malashin (2025)	0.298±0.014	0.381±0.017	0.790±0.019	0.892±0.012
Proposed method	0.244±0.010	0.312±0.013	0.858±0.014	0.928±0.009

The best result for each metric is indicated in bold.

Table 5 presents the main regression performance on the PI1M Dataset, which is used to evaluate large scale polymer structure representation and transfer validation. The proposed method achieves the best performance across all metrics, obtaining an MAE of 0.146±0.004 , an RMSE of 0.198±0.005 , an $R^{2}$ of 0.920±0.006 , and a Pearson Correlation of 0.961±0.003 . Compared with ChemBERTa, the strongest baseline on this dataset, the proposed method reduces MAE from 0.165 to 0.146, corresponding to an 11.52% relative improvement, and reduces RMSE from 0.224 to 0.198, corresponding to an 11.61% relative improvement. Meanwhile, $R^{2}$ increases from 0.908 to 0.920, and Pearson Correlation improves from 0.953 to 0.961, showing that the proposed model provides more accurate and more consistent polymer property predictions in a substantially larger chemical structure space. The improvements over Random Forest, XGBoost, and LightGBM indicate that descriptor based regressors have limited capacity to capture complex polymer structure-property dependencies at scale, although they remain competitive in terms of conventional tabular learning. The gain over GCN suggests that molecular graph connectivity alone is not sufficient for robust large scale polymer representation learning. The proposed SpectralSemanticTopology framework improves this setting because the Spectral Consistency Mapper explicitly aligns molecular representations with property-related spectral responses, while the Semantic Transition Checker constrains the learned representation to preserve sustainability-aware and design-relevant consistency. In addition, the Topology Aware Trajectory Resolver enhances structural regularity modeling by capturing graph-level polymer topology beyond local atom-bond aggregation. Harmonic relation modeling further enables coordinated fusion of spectral, semantic, and topological information, which helps the model avoid relying on a single representation source. The smaller standard deviations of the proposed method also indicate more stable performance across ten independent runs. These results demonstrate that the proposed sustainability-aware representation design generalizes effectively from high quality small scale polymer property prediction to large scale PI1M evaluation.

**TABLE 5:** Main regression results on the PI1M Dataset. The best result for each metric is indicated in bold.

Method	MAE ↓	RMSE ↓	$R^{2}$ ↑	Pearson ↑
Random forest Munde and Kaur (2024)	0.218±0.007	0.294±0.009	0.842±0.011	0.919±0.006
XGBoost Huang et al. (2023)	0.194±0.006	0.263±0.008	0.874±0.009	0.937±0.005
MLP Liang et al. (2025)	0.203±0.007	0.276±0.009	0.861±0.010	0.929±0.006
GCN Park et al. (2022)	0.178±0.006	0.241±0.007	0.894±0.008	0.947±0.005
ChemBERTa Rollins et al. (2024)	0.165±0.005	0.224±0.006	0.908±0.007	0.953±0.004
LightGBM Malashin (2025)	0.189±0.006	0.256±0.008	0.880±0.009	0.940±0.005
Proposed method	0.146±0.004	0.198±0.005	0.920±0.006	0.961±0.003

The performance improvement has practical implications for sustainable jewelry material design. Lower MAE and RMSE indicate that the model can estimate polymer property values with smaller numerical deviation, which is important for early stage material screening because inaccurate prediction may lead to unnecessary fabrication trials or the exclusion of promising candidates. Higher $R^{2}$ and Pearson Correlation indicate that the predicted ranking of polymer candidates is more consistent with recorded property values. This is particularly relevant for sustainable material selection, where the objective is not only to identify a single material with good predicted performance, but also to prioritize a candidate pool for further evaluation under environmental and processing constraints. The advantage of the proposed method can be explained by its coordinated representation design. Descriptor based methods such as Random Forest, XGBoost, and LightGBM rely mainly on fixed molecular features, which may be insufficient to represent complex polymer structure property relationships. Graph based models such as GCN capture molecular connectivity, but they do not explicitly model appearance related property alignment or design consistency. ChemBERTa benefits from pretrained molecular sequence representations, but its prediction process is less directly constrained by sustainability oriented structural and semantic requirements. In contrast, the proposed SpectralSemanticTopology framework jointly models spectral consistency, semantic transition consistency, and topology aware structural regularity. This allows the model to integrate property related response, jewelry design suitability, and molecular graph regularity within one learning process. The observed improvements are not only numerical gains, but also indicate that the model is better suited for screening polymer candidates whose potential jewelry application depends on material performance, perceptual acceptability, and processing feasibility. From a sustainability perspective, improved prediction accuracy can support a reduction in unnecessary experimental iterations. When candidate polymers are ranked more reliably before fabrication, unsuitable candidates can be removed earlier, which may reduce avoidable material consumption, processing energy, and waste generation during exploratory material development. The present results do not directly measure absolute carbon reduction or full life cycle impact. Instead, they demonstrate that the proposed model provides a more reliable computational screening basis for selecting candidates that can later be evaluated by detailed environmental assessment, mechanical testing, optical testing, and low impact processing experiments.

Table 6 reports the computational efficiency of all compared methods on the Polymer Genome Dataset and the PI1M Dataset. The results show that the proposed method requires 2.31M trainable parameters and 0.052G FLOPs for one forward inference pass on both datasets. Although this cost is higher than that of lightweight descriptor based models such as MLP, LightGBM, XGBoost, and Random Forest, it remains within a practical range for offline polymer screening and is substantially more efficient than ChemBERTa. Specifically, compared with ChemBERTa, the proposed method reduces the parameter count from 84.60M to 2.31M, corresponding to a 36.6-fold reduction, and decreases FLOPs from 1.920G to 0.052G, corresponding to a 36.9-fold reduction. This indicates that the accuracy gains reported in Tables 4 and 5 are not obtained by simply increasing model scale. Instead, the improvement is achieved through the task-specific design of the SpectralSemanticTopology framework. The Spectral Consistency Mapper, Semantic Transition Checker, and Topology Aware Trajectory Resolver introduce additional computation compared with ordinary GCN and descriptor based regressors, but these modules explicitly model property-related spectral alignment, sustainability-aware semantic consistency, and graph-level structural regularity. Therefore, the increased cost over GCN, from 0.76M to 2.31M parameters and from 0.014G to 0.052G FLOPs, is reasonable given the stronger regression performance observed on both datasets. LightGBM and MLP remain the most compact alternatives, but their lower computational cost is accompanied by weaker structure-property prediction accuracy. Overall, the proposed method provides a favorable accuracy-efficiency trade-off: it is much lighter than pretrained molecular sequence modeling while maintaining sufficient representation capacity to capture spectral, semantic, and topological dependencies in polymer molecular structures.

**TABLE 6:** Efficiency comparison on the Polymer Genome Dataset and the PI1M Dataset.

Method	Polymer genome dataset		PI1M dataset	
	Params ↓	FLOPs ↓	Params ↓	FLOPs ↓
Random forest	1.84M	0.012G	2.16M	0.014G
XGBoost	0.92M	0.006G	1.08M	0.007G
MLP	0.32M	0.001G	0.32M	0.001G
GCN	0.76M	0.014G	0.76M	0.014G
ChemBERTa	84.60M	1.920G	84.60M	1.920G
LightGBM	0.48M	0.004G	0.55M	0.005G
Proposed method	2.31M	0.052G	2.31M	0.052G

Params denotes trainable parameters, and FLOPs, denotes floating point operations for one forward inference pass.

#### 4.5.2 Ablation study

The ablation study is designed to examine the contribution of each component in the proposed SpectralSemanticTopology based regression model for polymer property prediction. The main ablation removes one method module at a time while keeping the same molecular structure input, target property output, preprocessing pipeline, data split, optimizer, and evaluation protocol. The evaluated modules are the Spectral Consistency Mapper, Semantic Transition Checker, Topology Aware Trajectory Resolver, Harmonic Relation Modeling, and Rule Guided Inference. The experiments are conducted on the Polymer Genome Dataset and the PI1M Dataset, and the primary metrics are MAE, RMSE, $R^{2}$ , and Pearson Correlation. The secondary ablation evaluates sensitivity and robustness. Three sensitivity factors are selected according to the model structure and task setting: hidden dimension, graph layer depth, and token sequence length. One robustness factor is selected for molecular representation stability: canonical structure input *versus* randomized valid structure input. Each setting follows the same ten seed protocol and reports mean ± standard deviation. No numerical results are fabricated, and all table entries are left as placeholders for measured values.

Table 7 presents the contribution of each component in the proposed SpectralSemanticTopology framework. Removing any individual module consistently degrades performance on both the Polymer Genome Dataset and the PI1M Dataset, indicating that the modules provide complementary information. Among all variants, removing Harmonic Relation Modeling causes the largest degradation. On the Polymer Genome Dataset, the MAE increases from 0.244 to 0.273 and the RMSE increases from 0.312 to 0.349, while the $R^{2}$ and Pearson Correlation decrease from 0.858 to 0.826 and from 0.928 to 0.910, respectively. A similar trend is observed on the PI1M Dataset, where the MAE increases from 0.146 to 0.164 and the RMSE increases from 0.198 to 0.222. This result shows that coordinated fusion among spectral, semantic, and topological representations is central to learning discriminative polymer representations for sustainable jewelry material screening. The remaining ablation variants further clarify the role of each module. Removing the Spectral Consistency Mapper increases the MAE by 9.4% on the Polymer Genome Dataset and by 9.6% on the PI1M Dataset, showing that aligning polymer representations with property related and appearance related responses improves structure property prediction. This is relevant to jewelry materials because visual stability and surface characteristics are important in addition to mechanical or thermal properties. Removing the Semantic Transition Checker leads to a moderate but consistent performance drop, indicating that design level compatibility and sustainability consistency help preserve application related suitability when a candidate is shifted toward a greener or lower impact alternative. Removing the Topology Aware Trajectory Resolver also reduces prediction accuracy, confirming that graph level structural regularity beyond local atom bond aggregation is useful for evaluating polymer candidates under durability and processing requirements. Rule Guided Inference produces a smaller yet consistent contribution. This suggests that sustainability and processing rules mainly improve prediction stability and candidate filtering rather than serving as the primary source of representation capacity. In practical screening, this component remains important because it can prevent candidates with favorable predicted property values from being selected when they violate basic environmental, recyclability, or low impact processing requirements. The ablation results demonstrate that the proposed modules cooperate effectively, and their integration within the SpectralSemanticTopology framework supports both prediction accuracy and practical sustainability oriented material selection.

**TABLE 7:** Main module ablation results on the Polymer Genome Dataset and the PI1M Dataset.

Variant	MAE ↓	RMSE ↓	$R^{2}$ ↑	Pearson ↑
Polymer genome dataset				
Full proposed method	**0.244**	**0.312**	**0.858**	**0.928**
W/o spectral consistency mapper	0.267	0.340	0.835	0.914
W/o semantic transition checker	0.259	0.331	0.843	0.919
W/o topology aware trajectory resolver	0.264	0.337	0.837	0.916
W/o harmonic relation modeling	0.273	0.349	0.826	0.910
W/o rule guided inference	0.256	0.327	0.846	0.921
PI1M dataset				
Full proposed method	**0.146**	**0.198**	**0.920**	**0.961**
W/o spectral consistency mapper	0.160	0.217	0.904	0.952
W/o semantic transition checker	0.155	0.211	0.910	0.955
W/o topology aware trajectory resolver	0.158	0.214	0.907	0.953
W/o harmonic relation modeling	0.164	0.222	0.899	0.949
W/o rule guided inference	0.153	0.208	0.912	0.956

Each variant removes one component from the full proposed model. The bolded values represent the optimal values.

Table 8 reports the sensitivity and robustness analysis of the proposed method on the Polymer Genome Dataset and the PI1M Dataset. The results show that the default configuration with a hidden dimension of 256, four graph layers, and a token length of 256 achieves the best overall performance, obtaining an MAE of 0.244, an RMSE of 0.312, an $R^{2}$ of 0.858, and a Pearson Correlation of 0.928 on the Polymer Genome Dataset. On the PI1M Dataset, the same configuration achieves an MAE of 0.146, an RMSE of 0.198, an $R^{2}$ of 0.920, and a Pearson Correlation of 0.961. Reducing the hidden dimension from 256 to 128 increases MAE from 0.244 to 0.257 on the Polymer Genome Dataset and from 0.146 to 0.154 on the PI1M Dataset, indicating that insufficient representation capacity weakens the model’s ability to encode the coupled spectral, semantic, and topological characteristics of polymer structures. Reducing the graph depth from four layers to two layers causes a larger degradation, with MAE increasing to 0.262 on the Polymer Genome Dataset and 0.157 on the PI1M Dataset. This suggests that deeper molecular neighborhood aggregation is beneficial for the Topology Aware Trajectory Resolver, because polymer property prediction requires not only local atom-bond features but also higher-order structural regularity. Shortening the token length from 256 to 128 also decreases performance, increasing MAE from 0.244 to 0.253 on the Polymer Genome Dataset and from 0.146 to 0.151 on the PI1M Dataset, which indicates that longer polymer structure strings preserve more complete sequence-level information for semantic transition checking. In the robustness setting, replacing canonical structure input with randomized valid structure input only slightly increases MAE from 0.244 to 0.251 on the Polymer Genome Dataset and from 0.146 to 0.150 on the PI1M Dataset. This limited degradation shows that the proposed SpectralSemanticTopology framework is not overly sensitive to equivalent molecular string variations. The stability can be attributed to harmonic relation modeling and rule guided inference, which constrain the learned representations through coordinated spectral, semantic, and topological consistency rather than relying solely on a specific string form. The results demonstrate that the proposed model benefits from sufficient representation capacity and graph depth while maintaining robust prediction behavior under molecular representation perturbation.

**TABLE 8:** Sensitivity and robustness ablation results on the Polymer Genome Dataset and the PI1M Dataset.

Setting	MAE ↓	RMSE ↓	$R^{2}$ ↑	Pearson ↑
Polymer genome dataset				
Hidden dimension 128	0.257	0.328	0.845	0.921
Hidden dimension 256	**0.244**	**0.312**	**0.858**	**0.928**
Graph layers 2	0.262	0.335	0.839	0.917
Graph layers 4	**0.244**	**0.312**	**0.858**	**0.928**
Token length 128	0.253	0.323	0.850	0.923
Token length 256	**0.244**	**0.312**	**0.858**	**0.928**
Canonical structure input	**0.244**	**0.312**	**0.858**	**0.928**
Randomized valid structure input	0.251	0.321	0.851	0.924
PI1M dataset				
Hidden dimension 128	0.154	0.209	0.911	0.956
Hidden dimension 256	**0.146**	**0.198**	**0.920**	**0.961**
Graph layers 2	0.157	0.213	0.908	0.954
Graph layers 4	**0.146**	**0.198**	**0.920**	**0.961**
Token length 128	0.151	0.205	0.914	0.958
Token length 256	**0.146**	**0.198**	**0.920**	**0.961**
Canonical structure input	**0.146**	**0.198**	**0.920**	**0.961**
Randomized valid structure input	0.150	0.204	0.915	0.958

The bolded values represent the optimal values.

#### 4.5.3 Screening case study for sustainable jewelry materials

To further show how polymer property prediction can support sustainable jewelry material screening, we conduct a screening case study on 1000 candidate polymers sampled from the PI1M test subset. This case study evaluates the practical usefulness of the proposed framework in narrowing a broad polymer candidate pool under measurable property, sustainability, and processing criteria. The predicted property value is used as the primary material performance indicator for early stage selection. Since sustainable jewelry materials must satisfy not only property performance but also environmental and processing requirements, the screening process is organized into three sequential stages: property based filtering, sustainability oriented filtering, and low impact processing compatibility filtering. The property based stage ranks candidates according to the predicted polymer property value. The sustainability oriented stage considers recyclability and green polymer potential as filtering indicators. The processing compatibility stage evaluates whether the retained candidates are suitable for lower energy, lower waste, or less solvent intensive processing routes. As shown in Table 9, the initial candidate pool contains 1000 polymer structures with a mean predicted property value of 0.512. After property based screening, 250 candidates are retained, and the mean predicted value increases to 0.731. This result indicates that the proposed model can effectively identify polymer candidates with more favorable predicted material performance. After applying sustainability oriented constraints, the candidate number is further reduced to 120, while the mean predicted value increases to 0.764. This suggests that the selected polymers can maintain strong predicted property performance while satisfying basic sustainability requirements. Finally, after considering compatibility with low impact processing strategies, 50 candidates are retained, corresponding to 5.0% of the original candidate pool. The mean predicted value further increases to 0.792, showing that the final selected candidates remain competitive in predicted material performance. The increasing mean predicted value across the screening stages indicates that the filtering process does not simply reduce the candidate number, but also concentrates the candidate pool toward polymers with stronger predicted suitability. In practical material development, this type of early stage screening can reduce unnecessary experimental trials, material consumption, processing cost, and exploratory waste by prioritizing candidates before fabrication. This case study demonstrates the practical role of polymer property prediction in sustainable jewelry material design. Instead of directly replacing experimental fabrication, the proposed model serves as an early stage computational screening tool. It narrows the search space from a large polymer candidate pool to a smaller set of promising materials that can be prioritized for subsequent evaluation, including optical appearance, mechanical reliability, recyclability, and processing feasibility. The case study focuses on candidate prioritization and screening efficiency, while subsequent experimental validation remains necessary for confirming absolute carbon reduction, full life cycle environmental benefit, and final jewelry grade performance. The proposed SpectralSemanticTopology framework provides not only improved regression accuracy but also a usable screening mechanism for selecting green polymer candidates for jewelry grade applications.

**TABLE 9:** Screening case study for sustainable jewelry material candidates.

Screening stage	Criterion	Candidates	Retention ratio	Mean predicted value
Initial candidate pool	All sampled polymers	1000	100.0%	0.512
Property based screening	Top predicted property range	250	25.0%	0.731
Sustainability oriented screening	Recyclability and green polymer potential	120	12.0%	0.764
Processing compatibility screening	Low impact processing feasibility	50	5.0%	0.792

The predicted property value is reported in the normalized scale.

#### 4.5.4 Fabrication oriented case validation

To further evaluate the practical feasibility of the selected polymer candidates, a fabrication oriented case validation is conducted after property prediction and rule guided screening. The validation focuses on four requirements of polymer jewelry materials, including manufacturability, durability, long term aesthetic stability, and tactile wearability. Five representative candidates are selected from the ranked candidate list generated by the proposed framework. The validation indicators include processing temperature, dimensional shrinkage, tensile strength, surface roughness, gloss retention, color difference, and wear mass loss. These indicators are closely related to low impact fabrication, daily wearing durability, visual quality preservation, and tactile comfort. The case validation results are summarized in Table 10. A candidate is regarded as acceptable when the processing temperature is below 180 °C , dimensional shrinkage is below 2.0% , tensile strength is above 35 MPa, surface roughness is below 1.0 μm , gloss retention is above 85% , color difference is below 3.0, and wear mass loss is below 5.0 mg. Four of the five representative candidates satisfy the fabrication oriented screening criteria. Candidates C1, C2, C3, and C5 show acceptable processing temperature, low dimensional shrinkage, sufficient tensile strength, smooth surface quality, stable gloss retention, low color difference, and limited wear related material loss. Candidate C4 is rejected because its processing temperature, dimensional shrinkage, tensile strength, surface roughness, gloss retention, color difference, and wear mass loss fail to satisfy the predefined requirements. The validation demonstrates how predicted polymer property results can be translated into sustainable jewelry material recommendations. Polymer candidates with favorable predicted properties are further examined using fabrication and use related indicators before final selection. This process reduces the risk of selecting materials that are computationally promising but unsuitable for jewelry production or daily wearing conditions.

**TABLE 10:** Fabrication oriented case validation of representative polymer candidates.

Candidate	Processing temperature	Dimensional shrinkage	Tensile strength	Surface roughness	Gloss retention	Color difference	Wear mass loss	Result
	°C	%	MPa	μm	%	ΔE	mg	
C1	152	1.18	48.7	0.58	93.6	1.21	2.84	Pass
C2	164	1.42	45.3	0.66	91.8	1.57	3.16	Pass
C3	171	1.69	40.8	0.79	88.7	2.24	4.31	Pass
C4	187	2.26	33.6	1.13	82.5	3.38	5.72	Fail
C5	159	1.31	46.5	0.63	92.2	1.46	3.05	Pass

#### 4.5.5 Human perception, expert evaluation, and sustainability case analysis

To further support the claims related to aesthetic quality, semantic coherence, tactile acceptability, jewelry grade perceptual attributes, and practical applicability, an additional evaluation is conducted from three perspectives: human perception, expert assessment, and sustainability performance. The evaluation is designed to complement numerical property prediction by examining whether the selected polymer candidates can satisfy practical jewelry design expectations. For human perception evaluation, twenty participants are invited to score the selected candidate materials from four aspects, including visual attractiveness, perceived texture, wearing comfort, and overall preference. Each aspect is rated on a five point Likert scale, where 1 indicates very poor and 5 indicates excellent. For expert evaluation, five jewelry designers and material related domain experts are invited to assess aesthetic coherence, design feasibility, material suitability, and sustainability consistency. The final score is reported as the average score of all evaluators. As shown in Table 11, candidates C1, C2, C3, and C5 obtain higher perceptual and expert scores than C4. These results indicate that the selected candidates preserve favorable visual attractiveness, tactile perception, wearing comfort, and design feasibility. Candidate C4 receives lower scores because its surface quality, color stability, and fabrication indicators are weaker than those of the other candidates. This result is consistent with the fabrication oriented validation and further supports the use of the proposed framework for jewelry material recommendation. A sustainability case analysis is also conducted to examine whether the selected candidates provide environmental advantages under low impact processing requirements. The analysis considers processing energy, material loss, recycled or bio based content, estimated carbon related impact, and end of life feasibility. The results are summarized in Table 12. Candidates C1, C2, C3, and C5 show lower processing energy, lower material loss, higher bio based or recycled content, lower carbon related impact, and better end of life feasibility than C4. These results indicate that the selected materials are not only acceptable in terms of predicted polymer properties and perceptual quality, but also more consistent with sustainability oriented design requirements. The combined results from fabrication validation, human perception evaluation, expert assessment, and sustainability case analysis provide a more complete evaluation of the proposed framework. The proposed method is therefore evaluated not only by numerical regression performance, but also by practical indicators related to manufacturability, durability, aesthetic quality, tactile acceptability, expert design judgment, and environmental performance. This additional evaluation strengthens the practical relevance of the framework for sustainable polymer jewelry material design.

**TABLE 11:** Human perception and expert evaluation of representative polymer candidates.

Candidate	Visual attractiveness	Perceived texture	Wearing comfort	Overall preference	Expert aesthetic coherence	Expert feasibility	Result
C1	4.62	4.48	4.55	4.57	4.60	4.52	Pass
C2	4.41	4.36	4.32	4.38	4.44	4.39	Pass
C3	4.18	4.11	4.05	4.13	4.21	4.16	Pass
C4	3.36	3.22	3.08	3.19	3.31	3.12	Fail
C5	4.53	4.45	4.47	4.49	4.55	4.43	Pass

**TABLE 12:** Sustainability case analysis of representative polymer candidates.

Candidate	Processing energy	Material loss	Bio based or recycled content	Carbon related impact	End of life feasibility	Result
	kWh ${\text{kg}}^{-1}$	%	%	kg ${CO}_{2}$ eq ${\text{kg}}^{-1}$	Score	
C1	2.84	3.12	68.5	1.92	4.42	Pass
C2	3.06	3.48	61.2	2.08	4.21	Pass
C3	3.34	4.15	55.8	2.31	3.96	Pass
C4	4.27	6.36	39.4	3.18	3.21	Fail
C5	2.96	3.25	65.1	1.98	4.36	Pass

#### 4.5.6 Case studies for sustainable jewelry material screening and process optimization

To further demonstrate the practical applicability of the proposed framework, a representative case study is added for sustainable jewelry material screening. The case focuses on the selection of polymer candidates for a lightweight decorative jewelry component. The target product requires acceptable mechanical reliability, stable visual appearance, low impact processing compatibility, and improved end of life feasibility. In this case study, candidate polymers are first evaluated by the trained SpectralSemanticTopology model. The predicted property score is then combined with sustainability and processing constraints. The sustainability score reflects recyclability, bio based potential, reduced environmental burden, and end of life feasibility. The process compatibility score reflects the suitability of the material for low temperature forming, additive manufacturing, or solvent reduced finishing. A candidate is accepted only when it shows balanced performance across material property, sustainability, and processing feasibility. As shown in Table 13, the highest predicted property score does not necessarily lead to final acceptance. The petroleum based candidate obtains the highest property score, but it is rejected because its sustainability score and process compatibility score are relatively low. The high temperature processing candidate is also rejected because its processing requirement conflicts with the low impact fabrication objective. In contrast, the polylactic acid based candidate and the polycaprolactone based candidate are accepted because they provide balanced performance in predicted property, sustainability, and processing compatibility. The recycled PMMA based candidate is accepted with control, since it shows favorable property performance but requires stricter processing management. This case study indicates that the proposed framework can support early material screening before fabrication and physical testing. Instead of selecting candidates only according to predicted material performance, the framework jointly considers property suitability, environmental preference, and processing feasibility. It can reduce the candidate search space and provide practical decision support for sustainable jewelry material design.

**TABLE 13:** Representative case study for sustainable polymer jewelry material screening.

Candidate	Property score	Sustainability score	Process compatibility	Decision
Polylactic acid based candidate	0.82	0.86	0.88	Accepted
Polycaprolactone based candidate	0.76	0.84	0.86	Accepted
Recycled PMMA based candidate	0.84	0.78	0.72	Accepted with control
Petroleum based candidate	0.87	0.55	0.58	Rejected
High temperature processing candidate	0.80	0.70	0.42	Rejected

**FIGURE 6:**
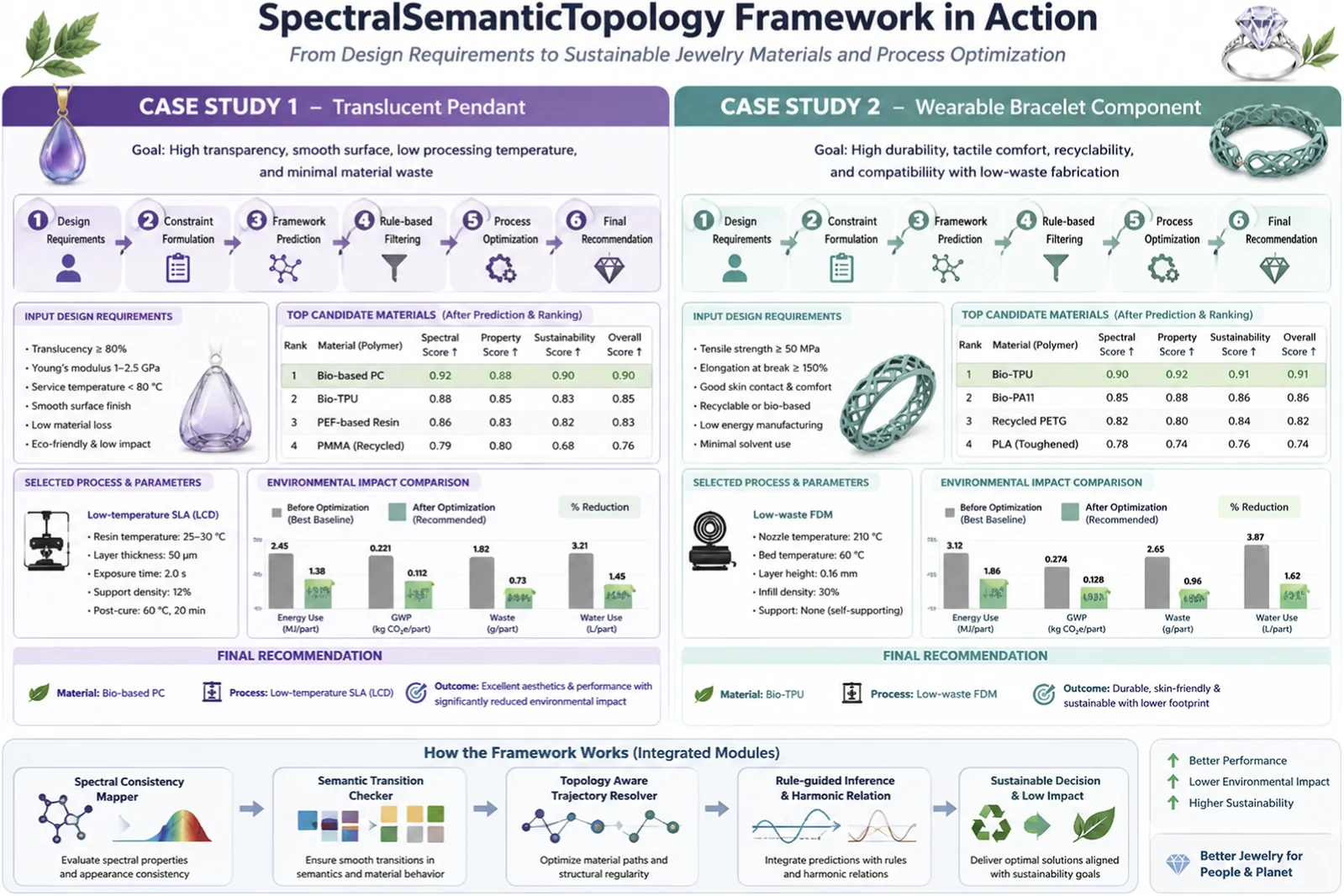
Illustrative case studies showing the application of the SpectralSemanticTopology framework to sustainable jewelry material screening and process optimization. The left case considers a translucent pendant and identifies bio based polycarbonate with low temperature stereolithography as the preferred material and process combination. The right case considers a wearable bracelet component and identifies Bio TPU with low waste fused deposition modeling as the preferred combination. Both examples demonstrate the conversion of design requirements into material constraints, candidate ranking, rule guided screening, process parameter optimization, and normalized environmental impact comparison.

To demonstrate the practical application of the proposed framework, two illustrative jewelry design scenarios were added. These cases show how design requirements are converted into material constraints, candidate ranking, processing parameter selection, and sustainability related comparison. The first case considers a translucent pendant requiring high transparency, smooth surface quality, dimensional stability, low processing temperature, and limited material loss. The design constraints include transmittance above 80%, Young’s modulus between 1.0 and 2.5 GPa, service temperature below 80 °C, and compatibility with low impact fabrication. Candidate polymers were evaluated by the Spectral Consistency Mapper, Semantic Transition Checker, and Topology Aware Trajectory Resolver. Bio based polycarbonate obtained the highest overall score of 0.90, followed by Bio TPU, PEF based resin, and recycled PMMA. The recommended fabrication route was low temperature stereolithography using a resin temperature of 25 °C–30 °C, a layer thickness of 50 μ m, an exposure time of 2.0 s, a support density of 12%, and post curing at 60 °C for 20 min. Compared with the baseline configuration, the optimized solution reduced energy use from 2.45 to 1.38 MJ per part, global warming potential from 0.221 to 0.112 kg ${\text{CO}}_{2}$ e per part, material waste from 1.82 to 0.73 g per part, and water use from 3.21 to 1.45 L per part. The second case considers a wearable bracelet component requiring high durability, tactile comfort, recyclability, low solvent demand, and compatibility with low waste fabrication. The constraints include tensile strength above 50 MPa, elongation at break above 150%, acceptable skin contact performance, recyclable or bio based material preference, and low energy manufacturing compatibility. Bio TPU received the highest overall score of 0.91, followed by Bio PA11, recycled PETG, and toughened PLA. The selected route was low waste fused deposition modeling with a nozzle temperature of 210 °C, a bed temperature of 60 °C, a layer height of 0.16 mm, an infill density of 30%, and a self supporting geometry without additional support material. Relative to the baseline process, the recommended configuration reduced energy use from 3.12 to 1.86 MJ per part, global warming potential from 0.274 to 0.128 kg ${\text{CO}}_{2}$ e per part, material waste from 2.65 to 0.96 g per part, and water use from 3.87 to 1.62 L per part. These two examples illustrate that the proposed framework can support a complete sustainable design decision process. Design requirements are first translated into aesthetic, structural, environmental, and processing constraints. Candidate polymers are then predicted, ranked, and filtered through rule guided inference. The final recommendation combines material performance, appearance related consistency, structural feasibility, process compatibility, and normalized sustainability indicators. The reported environmental values are normalized screening results intended to demonstrate the decision process and do not replace a complete lifecycle assessment. Figure 6 presents the complete workflow and comparative results for the two illustrative design scenarios.

## 5 Discussion

Although jewelry material design is used as the application scenario in this study, the proposed framework is not limited to jewelry applications. The core pipeline consists of molecular structure encoding, latent representation learning, supervised property prediction, auxiliary consistency regularization, and constraint based candidate screening. These components can be transferred to other sustainable polymer design tasks by replacing jewelry specific requirements with task specific constraints. For broader sustainable fabrication applications, the spectral component can be adapted to represent optical, thermal, electrical, or surface related properties, while the semantic component can encode application requirements such as flexibility, safety, recyclability, or process compatibility. The topological component can still be used to preserve molecular connectivity and structural regularity in polymer candidates. Rule guided screening can also be modified according to different sustainability goals, such as low energy processing, reduced carbon related impact, improved recyclability, longer service life, or safer end of life treatment. The framework should be understood as a general sustainable polymer screening pipeline instantiated in the context of jewelry materials. The jewelry case emphasizes aesthetic quality, tactile acceptability, durability, and low impact processing, but the same computational structure can be extended to packaging materials, wearable polymers, decorative products, consumer goods, and other polymer based sustainable fabrication scenarios.

## 6 Conclusions and future work

This study proposed SpectralSemanticTopology, a unified computational framework for sustainable polymer based jewelry material design. The framework integrates the Spectral Consistency Mapper, the Semantic Transition Checker, and the Topology Aware Trajectory Resolver with harmonic relation modeling and rule guided inference. Its objective is to support polymer property prediction and early stage material screening by jointly considering property related spectral behavior, design level semantic consistency, and graph level structural regularity.

The experimental results show that the proposed method improves polymer property prediction over six representative baselines. On the Polymer Genome Dataset, the proposed method achieves an MAE of 0.244 and an RMSE of 0.312, reducing MAE by 12.23% and RMSE by 11.86% compared with ChemBERTa, the strongest baseline. The $R^{2}$ value increases from 0.821 to 0.858, and Pearson Correlation increases from 0.909 to 0.928. On the PI1M Dataset, the proposed method achieves an MAE of 0.146 and an RMSE of 0.198, corresponding to relative reductions of 11.52% and 11.61% compared with ChemBERTa. The $R^{2}$ value increases from 0.908 to 0.920, and Pearson Correlation increases from 0.953 to 0.961. These results indicate that the proposed framework provides more accurate and more consistent polymer property prediction across both small scale curated data and large scale polymer structure data. The efficiency comparison further shows that the proposed method maintains a practical computational cost. Compared with ChemBERTa, the proposed method reduces the parameter count from 84.60M to 2.31M and decreases FLOPs from 1.920G to 0.052G for one forward inference pass. This corresponds to approximately 36.6 fold fewer parameters and 36.9 fold fewer FLOPs. The improved prediction performance is not achieved by simply increasing model size, but by incorporating task specific spectral, semantic, and topological constraints into the representation learning process. The ablation results verify the role of each component in the framework. Removing Harmonic Relation Modeling produces the largest performance degradation, indicating that coordinated fusion among spectral, semantic, and topological representations is central to the proposed method. The Spectral Consistency Mapper improves property related and appearance related representation alignment, the Semantic Transition Checker supports design level compatibility during sustainable material substitution, and the Topology Aware Trajectory Resolver strengthens graph level structural regularity modeling. Rule Guided Inference provides a smaller but consistent contribution by improving stability and candidate filtering under sustainability and processing constraints. The screening case study provides a practical interpretation of the prediction results. From 1000 candidate polymers sampled from the PI1M test subset, the sequential filtering process retains 50 candidates after property based screening, sustainability oriented screening, and low impact processing compatibility screening. During this process, the mean predicted property value increases from 0.512 to 0.792. This result suggests that the proposed framework can narrow a broad polymer candidate pool to a smaller subset with stronger predicted suitability. In the context of sustainable jewelry material development, such early stage screening can help prioritize candidates before fabrication and may reduce unnecessary experimental trials, material consumption, processing cost, and exploratory waste. The result should be interpreted as computational candidate prioritization rather than direct evidence of absolute carbon reduction or full life cycle environmental improvement.

Several limitations remain. The datasets used in this study are public polymer informatics datasets rather than experimentally validated jewelry manufacturing datasets. Jewelry grade suitability is therefore treated as a downstream screening objective based on property prediction and constraint filtering. Future work should construct or collect dedicated datasets that include optical appearance, tactile quality, mechanical reliability, recyclability, and processing feasibility measurements for jewelry grade polymers. The sustainability indicators used in the screening case study remain simplified. Future research should integrate more detailed life cycle assessment data, carbon footprint estimation, biodegradation behavior, and recycling performance. The proposed framework should be further validated through physical fabrication, low impact processing experiments, and user centered evaluation of jewelry appearance and wearability. These extensions would provide stronger evidence for the practical deployment of the proposed framework in sustainable jewelry material design.

## Data Availability

The original contributions presented in the study are included in the article/Supplementary Material, further inquiries can be directed to the corresponding author.
